# Desiccation-Driven Senescence in the Resurrection Plant *Xerophyta schlechteri* (Baker) N.L. Menezes: Comparison of Anatomical, Ultrastructural, and Metabolic Responses Between Senescent and Non-Senescent Tissues

**DOI:** 10.3389/fpls.2019.01396

**Published:** 2019-10-30

**Authors:** Astrid Lillie Radermacher, Stephanus Francois du Toit, Jill M. Farrant

**Affiliations:** Department of Molecular and Cell Biology, University of Cape Town, Cape Town, South Africa

**Keywords:** age-related senescence, desiccation tolerance, resurrection plant, senescence prevention, *Xerophyta schlechteri*

## Abstract

Drought-induced senescence is a degenerative process that involves the degradation of cellular metabolites and photosynthetic pigments and uncontrolled dismantling of cellular membranes and organelles. Angiosperm resurrection plants display vegetative desiccation tolerance and avoid drought-induced senescence in most of their tissues. Developmentally older tissues, however, fail to recover during rehydration and ultimately senesce. Comparison of the desiccation-associated responses of older senescent tissues (ST) with non-ST (NST) will allow for understanding of mechanisms promoting senescence in the former and prevention of senescence in the latter. In the monocotyledonous resurrection plant *Xerophyta schlechteri* (Baker) N.L. Menezes*, leaf tips senesce following desiccation, whereas the rest of the leaf blade survives. We characterized structural and metabolic changes in ST and NST at varying water contents during desiccation and rehydration. Light and transmission electron microscopy was used to follow anatomical and subcellular responses, and metabolic differences were studied using gas chromatography-mass spectrometry and colorimetric metabolite assays. The results show that drying below 35% relative water content (0.7 gH_2_O/g dry mass) in ST resulted in the initiation of age-related senescence hallmarks and that these tissues continue this process after rehydration. We propose that an age-related desiccation sensitivity occurs in older tissues, in a process metabolically similar to that observed during age-related senescence in *Arabidopsis thaliana*.

## Introduction

Resurrection plants are a rare subset of plant species capable of desiccation tolerance in vegetative tissues. Desiccation tolerance is the ability to sustain loss of up to 95% of subcellular water, to engage prolonged quiescence in the dry state, and to revive full metabolic activity within hours to days following rehydration (Gaff 1977). Research in recent years has positioned resurrection plants as unique models for understanding the mechanisms required for mitigating extensive water deficit stress and providing insight as to mechanisms for improvement of drought tolerance strategies in crops (Farrant et al., 2015; Hilhorst and Farrant, 2018). This will be increasingly important for food security in an ever-warming world.

*Xerophyta schlechteri* ^1^ (Baker) N.L. Menezes is one of the most widely studied poikilochlorophyllous monocotyledonous angiosperm resurrection plants. Like other poikilochlorophyllous resurrection plants, *X. schlechteri* minimizes photo-oxidative stress and reactive oxygen species (ROS) formation during drying by breaking down chlorophyll and thylakoid membranes (Sherwin and Farrant, 1998; Mundree and Farrant, 2000; Farrant et al., 2015). Biochemically, it employs an early (prior to shutdown of photosynthetic activity) and late (below *ca*. 55% RWC) response to drying, differentially engaging molecular chaperones, translational machinery, signal transducers, and gene regulators to alter metabolism, respiration, photosynthesis, and organelle structure to bring about quiescence (Farrant et al., 2015).

In desiccation sensitive plants, including cereal crops, leaf senescence processes during drought are initiated to limit water loss through transpiration and to remobilize nutrients from older tissues toward younger tissues and reproductive organs, ultimately resulting in the death of the leaf (Woo et al., 2019). This is characterized by breakdown of chloroplasts, loss of photosynthetic pigments, protein degradation, nutrient remobilization (Munné-Bosch and Alegre 2004; Watanabe et al., 2013; Bresson et al., 2018), and autophagy (Woo et al., 2019). It has been proposed that resurrection plants are able to suppress drought-induced senescence (Griffiths et al., 2014; Williams et al., 2015) in the bulk of their tissues by employing signal blocking mechanisms. The same would be true of *X. schlechteri*, although the exact mechanisms associated with this are not yet known.

**Figure 1:**
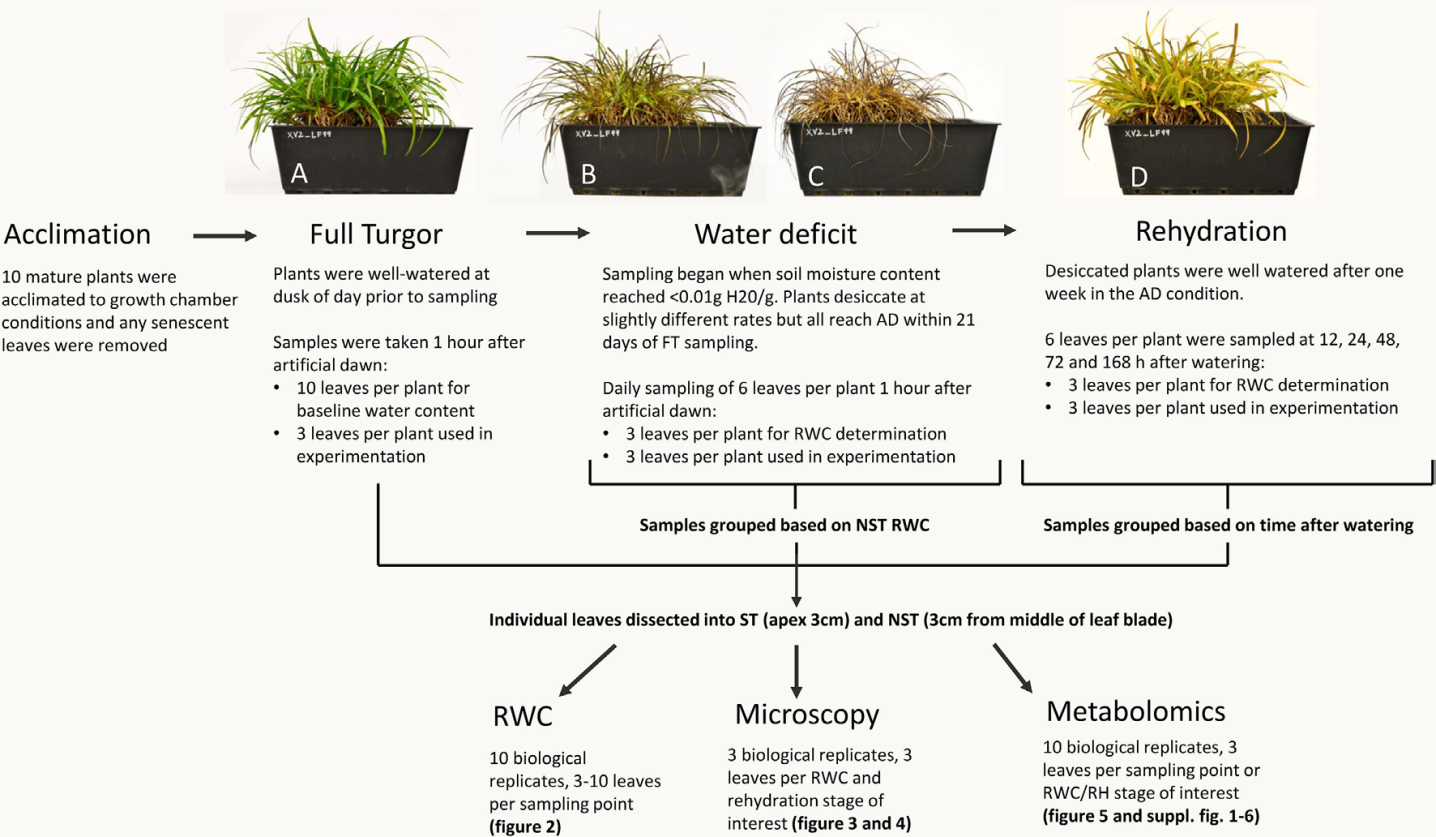
Sampling procedure for relative water content (RWC), microscopy, and metabolomics. Plants experiencing the same water deficit stress were sampled for multiple procedures. All 10 plants were utilized for water content determination (3–10 leaves per plant per sampling point) and metabolomics (3 leaves per plant per RWC/RH stage of interest), while microscopy was performed on three plants (3 leaves per plant per RWC/RH stage of interest). A typical experimental *X. schlechteri* is shown before, during, and after water deficit stress: FT **(A)**, 60% RWC **(B)**, AD **(C)**, and 48 h after rehydration **(D)**.

“Tip burning,” whereby the apex of the leaf fails to recover following desiccation, was observed in *X. schlechteri* in the present study (**Figure 1**). This led us to speculate that desiccation-driven senescence might be occurring in these tip tissues. To test this, we compared the anatomy, ultrastructure, and primary metabolism of the non-senescent tissues (NST) and senescent tissues (ST) of *X. schlechteri* during dehydration and rehydration, aiming to elucidate both the process of drought-induced senescence in ST and the mitigating features of senescence in NST. The presence of ST and NST on the same leaf allowed for changes in both tissue types to be studied in the context of a continuum. This is the first study to consecutively contrast the metabolic and physiological changes in NST and ST in this manner during the desiccation of a resurrection plant. Our findings provide insight into the mechanisms associated with the suppression of senescence in the bulk of the tissue in this species.

## Materials and Methods

### Experimental Conditions and Sampling Procedures

Mature *X. schlechteri* plants were collected from Buffelskloof Private Nature Reserve (25°19’48.83”S, 30°29’40.83”E) in the Mpumalanga province of South Africa and maintained in a greenhouse conditions as described in Sherwin and Farrant (1996). For the current experimentation, 10 mature plants were transferred to controlled environment chambers (Conviron Adaptis A350 Chamber, Canada) under the following conditions; 16 h light, 300 µmolm^−2^ s^−1^, 25°C; 8 h dark, 20°C. Plants were acclimated to these conditions for 1 week prior to induction of dehydration stress. Senesced leaf tips were removed from the plants 2 days into acclimation. Samples of fully hydrated (full turgor) plants were taken 8 h after artificial “dusk” on the last day of acclimation, therefore at artificial “dawn.” These were processed as described below. Dehydration was induced by cessation of soil watering. Once the soil moisture of each individual pot approached <0.01 g H_2_0/g, tissues were sampled at 1 h after artificial dawn. At each sampling point, three fully expanded leaves per plant were sampled and dissected to yield an apex region of 1 cm the ST and a 1-cm-length section of tissue from the middle of the leaf blade the NST. These were used to determine tissue relative water content (RWC) and for the concomitant anatomical, ultrastructural, and metabolite analyses. The sampling procedure is depicted in **Figure 1**. Once the plants had reached an air-dry state (RWC of NST leaves being <5%), they were maintained in the dry state for 7 days, after which they were rehydrated by watering the plants and soils. Tissues were sampled as above at 12, 24, 48, 72, and 168 h postrehydration.

### Relative Water Content Determination

The classic method of determination of plant RWC, as outlined by Barrs and Weatherley (1962), uses the formula:

(1) $$\begin{array}{c} \text{RWC (\%)=[(fresh weight-dry weight)/}\\ \text{ (turgid weight-dry weight)]}\times 100 \end{array}$$

Fresh and dry weights are determined before and after oven drying at 70°C for 48 h, and the absolute water content (AWC) is determined by subtraction of the former from the latter. The turgid weight represents the full turgor weight obtained by immersing the leaves in water in the dark for 24 h at 4°C.

As the leaf tissues of *X. schlechteri* do not absorb water during the overnight incubation, the turgid weight cannot be easily and accurately calculated. Thus, the AWC of NST tissues from fully hydrated plants, sampled at artificial dawn, was calculated (AWC_FTnst_) and used as the turgid weight in all subsequent water content determinations. Furthermore, preliminary assessment (data not shown) indicated that the AWC of the leaf tip tissue (ST) was lower than that of the mid-leaf tissues (NST). To monitor the water contents of NST and ST from the same leaf and at the same timepoints, we devised a modified formula for RWC determination.

Relative water content of NST at sampling point X:

(2) $$\%RWC_{NST}=\frac{AWC_{Xnst}}{AWC_{FTnst}}\times 100$$

Relative water content of ST at sampling point X:

(3) $$\%RWC_{ST}=\frac{AWC_{Xst}}{AWC_{FTnst}}\times 100$$

Thus, all RWC measures for ST are relative to FT NST. For comparative reasons, AWC are given in **Figure 1** and in the text where relevant. Leaf water contents were determined on all 10 plants at each sampling point.

### Light Microscopy

**Figure 2:**
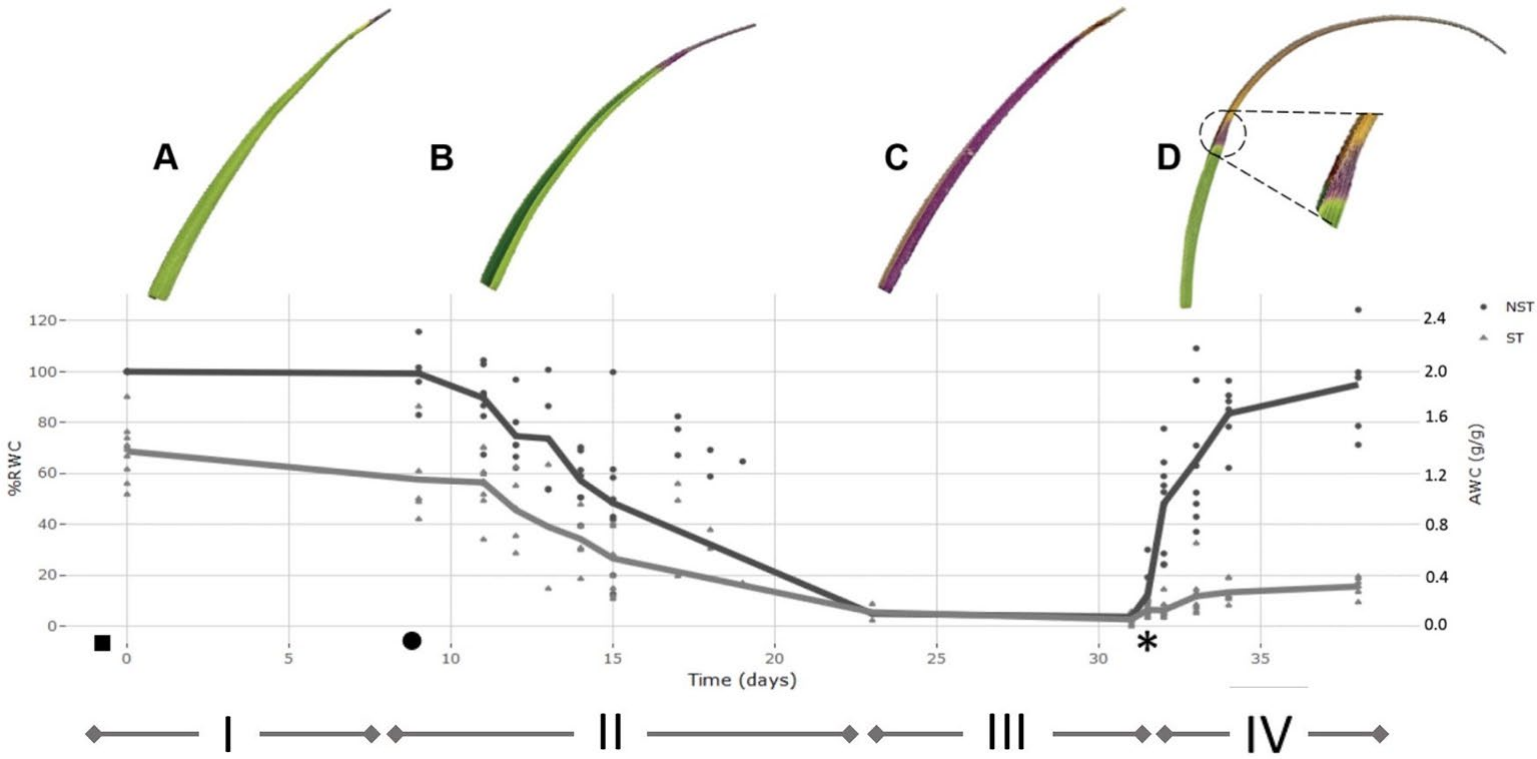
Desiccation-rehydration curve for the drying of *Xerophyta schlechteri*. The % relative water content (RWC) for 10 individuals was determined at daily intervals from the onset of water loss until the air-dry state was reached. The mean %RWC for each time point was used to plot the model dehydration curve. The model dehydration curve for both tissue types is shown [non-senescent tissues (NST) and ST]. All calculated values were set relative to the absolute water content of the NST at day 0 (100% RWC). The curve is divided into four distinct phases: i) fully hydrated; ii) slow dehydration of all tissues; iii) air-dry desiccated state; iv) rehydration after addition of water. The initial watering took place at dusk before day 0 (indicated with a ◻ ) and the first sampling at 1 h after dawn on day 0. When the soil moisture content was recorded as 0.01g H_2_0/g(◻), sampling for the desiccation phase was initiated. Plants were left in the dry state for one week, followed by rewatering at day 31 (*). High resolution scans of *X. schlechteri* leaves accompany each phase to illustrate physiological changes occurring in the leaf tissue, and to illustrate differential responses between the two tissue types. A represents FT, B represents 60% RWC in NST, C represents AD, D represents 72 h following rehydration. Triangle points are representative of ST and closed circles represent NST.

NST and ST tissues, from three representative plants, at daily sampling points were cut into sections and fixed in FAA fixative (formalin:acetic acid:95% ethanol, 10:5:50) for 24 h. Samples were serially dehydrated in a Leica TP1020 Tissue Processor with [50% (v/v) ethanol for 30 min, 70% (v/v) ethanol for 60 min, 95% (v/v) ethanol for 60 min; absolute ethanol for 60 min, three rounds of 6 h in absolute ethanol; and two rounds of 24 h paraffin wax infiltration at 60°C] before being embedded in paraffin wax. Sample blocks were sectioned to 10 µm using a Leica RM2125RT microtome and mounted on glass slides. Slides were dewaxed in xylol and then rehydrated through serial immersion in 100% (v/v), 90% (v/v), and 70% (v/v) ethanol. Each immersion lasted for 1 min and was performed in duplicate. Replicate slides were stained with 0.1% (w/v) Procion yellow in aqueous (2.5:1) dimethylformamide (DMF) for 60 min at 37°C (allows for visualization of chloroplasts and chloroplast aggregates) and then 0.1% (w/v) Alcian blue in 3% (v/v) acetic acid for 30 min at room temperature (allows for visualization of complex carbohydrates), with three 10 min washes in dH_2_O included after each staining. Slide staining methods were taken from protocols in Ruzin (1999). Glass coverslips were mounted on the stained slides with Mowiol mounting medium and allowed to air-dry for a minimum of 24 h, before being stored in slide boxes. Slides were investigated for changes in total tissue structure associated with the dehydration and rehydration for both NST and ST using a Nikon^®^ Eclipse Ti-E inverted Microscope. Only select representative samples at specific RWCs are shown in **Figure 2**.

### Transmission Electron Microscopy

Transmission electron microscopy (TEM) was used to investigate the ultrastructural details of the mesophyll cells from NST and ST at various stages of dehydration and rehydration. At each sampling point (FT, 75, 55, 35, and 5% RWC as well as 12, 24, 48, and 72 h post-rehydration), tissues from three plants were dissected into the respective tissue types (ST and NST), which were then fixed and processed for TEM in accordance with Cooper and Farrant (2002), with slight modifications. Briefly, small pieces of leaf tissue (approximately 2 mm^2^) were fixed in 2.5% glutaraldehyde in 0.1 m phosphate buffer (pH 7.4) containing 0.5% caffeine and then post-fixed in 1% osmium in phosphate buffer. After dehydration in a graded ethanol series, the tissues were immersed in 100% acetone, which was aspirated and replaced twice. The samples were incubated overnight at 4°C in one part acetone (500 µl) and one part Spurr’s resin (500 µl). Half of the solution was then removed and replaced with 500 µl Spurr’s resin to yield a 75% concentration of Spurr’s resin to acetone and incubated overnight at 4°C. This was repeated to yield an 87.5% solution. The solution was then removed and replaced with 100% Spurr’s resin and incubated overnight at 4°C. The samples were then placed into block moulds and allowed to harden at 60°C for 16 h. Samples were sectioned using a Richart Ultracut S Ultramicrotome. Sections (95 nm) were mounted on copper grids and stained with uranyl acetate (2% w/v) for 10 min and lead citrate (1% w/v, in NaOH chamber) for 10 min. Sections were viewed on a Tecnai T20 Electron Microscope.

### Metabolomics Sample Preparation

Leaves from 10 plants were immediately flash frozen, freeze dried and stored at −80°C until further analysis. For this analysis, tissues (5–15 mg) from each plant were dissected into their respective tissue types (NST and ST) and homogenized to a fine powder with a pestle and mortar. The powder was transferred to 2 ml centrifuge tubes. Residual material in the mortar was collected through the addition of 1 ml 80% (v/v) ethanol, which was added to the respective tubes. Samples were dried using vacuum centrifugation, and the mass of the dry powder starting material was determined. Metabolites were extracted using a modified phase separation extraction method described by Bieleski and Turner (1966). Modifications included filtration of all removed extracts through three layers of Whatman paper and concentration of the non-polar and polar fractions *via* lyophilization. These were used for the assays described below and gas chromatography–mass spectrometry analysis.

### Colorimetric Metabolite Assays

Dried non-polar fractions were resuspended in 500 µl 100% (v/v) acetone, 100 µl of which was then pipetted, in duplicate, into wells on a 96-well plate. The absorbance (*A*) of the extracts was measured at 662, 645, and 470 nm, using a Multiskan^™^ GO Microplate Spectrophotometer, and pigment concentrations were determined by using the equations of Lichtenthaler & Wellburn (1983). Metabolites were resuspended in 500 µl distilled water, and the sucrose, D-fructose, and D-glucose content determined using a R-Biopharm^®^ Sucrose/D-Glucose/D-Fructose Kit with the following modifications to the manufacturer’s protocol: absorbances were only taken at 340 nm (absorption maximum of NADPH) using a Multiskan^™^ GO Microplate Spectrophotometer. Initial data consolidation and formatting was conducted in Microsoft^®^ Excel Office 365. Subsequent data transformation and analysis were conducted in R version 3.3.3 using the packages “ggplot2” (Wickham, 2016) and “plotly” (Sievert, 2018). Metabolite data were normalized to the mass of the dry starting material. For statistical analysis, samples were divided into two data sets based on whether the starting material was collected during the dehydration or rehydration cycle. The points collected from the air-dry state (prior to rehydration) were used in both data sets to give continuity to the data. Points from the dehydration cycle had a negative value attributed to their %RWCs to give directionality, and both sets of data points were plotted on the same axis against %RWC. Independent log10 linear models were constructed for the two tissue types and these models were used to predict exponential models fitted to the raw data and were plotted on the same axis. Modeling information can be viewed in **Supplementary Table 1**.

### Gas Chromatography-Mass Spectrometry

Freeze-dried polar fractions were derivatized derivatized in methoxyamine hydrochloride and N-methyl-N-trimethylsilyltriflouroacetamide as described by (Lisec et al., 2006). A library of standards was prepared in the same manner using a mixture of amino acids (75 nmol each: alanine, arginine, aspartic acid, asparagine, glutamic acid, glycine, histidine, isoleucine, leucine, lysine, methionine, phenylalanine, proline, serine, threonine, ornithine, tryptophan, tyrosine, valine, cystine, and pyroglutamic acid). Sugar (10 ng xylose, arabinose, fucose, fructose, galactose, glucose, mannose, sucrose, cellobiose, maltose, myo-inositol, trehalose, raffinose), sugar alcohol (10 ng maltitol, sorbitol, galactitol, ribitol, xylitol, arabitol, erythritol) and tricarboxylic acid (TCA) cycle intermediate (10 ng succinic acid, fumaric acid, malic acid, and isocitric acid) standards were derivatized and run individually. Derivatized metabolite mixtures were analyzed on an Agilent Model 7890A Gas Chromatograph fitted with a 7693 Autosampler, interfaced with a 7000A Triple Quadrupole Mass Spectrometer (Agilent Technologies, Santa Clara, CA, USA). The helium flow was set to 1.39 ml/min. Samples of 1 µl were injected with a split ratio of 1:24 and resolved on a 30 m × 250 µm × 0.25 µm Agilent HP-5ms column. One technical repeat of each sample was run. A temperature program optimized for separation of sugars and sugar alcohols was utilized (Bartolozzi et al., 1997). Peak areas were calculated using OpenChrom^®^ Community Edition, and compounds were identified by comparison to the National Institute of Standards and Technology (NIST) Library (filtered for TMS derivatives) using the probability-based matching mass spectrum comparator with default parameters. Compound identification using the internal compound library was performed by aligning chromatograms to one another and this library using the GCAlignR package (Ottensmann et al., 2018) in R (v3.6.0) with default parameters. Peak area was normalized to the internal standard (ribitol) peak area and lyophilized sample mass. Comparative statistics (ANOVA with Fisher’s least significant difference method and t-test comparing relative abundance in tissue type) were performed in MetaboAnalyst 4.0 (Chong et al., 2018) using all identified and unidentified peaks, and heat maps were created in RStudio using the heatmap.2 function. Raw data is provided in **Supplementary Data Set 1**.

## Results

### Anatomical and Ultrastructural Changes During Dehydration and Rehydration

Under the conditions used in this work, the time taken for the leaf tissues of X. schlechteri to reach an air dry (desiccated) state after soil water depletion was 14 days (**Figures 2A, B**). This is similar to that reported by Farrant et al. (2015) to be typical of this species under such environmental conditions. The RWCs of the leaf tips, destined to become ST, were significantly lower (ca. 30–40%) than that of their matching mid-leaf NST counterparts throughout dehydration, until the desiccated state was reached in which both tissues stabilized at ≤3% RWC (**Figure 2C**). Upon rewatering, the RWC of the NST increased rapidly during the first 72 h, with tissues being fully hydrated by 1 week (**Figure 2D**). ST absorbed little water after rehydration, with the maximum calculated RWC being 20% (0.4 gH_2_O/g dry mass). High-resolution scans in **Figure 2** depict changes in the last 30% of the leaf blade during the different stages of dehydration and rehydration. In the fully hydrated state, the leaves were bright green with slightly yellowing apical tips (**Figure 2A**). During dehydration, the leaf blades folded along the midrib with the adaxial surfaces of the blades coming together, leaving only the abaxial surfaces exposed to the environment (**Figure 2B**). These become purple in color, indicating anthocyanin production, which has previously been reported for this species (Sherwin and Farrant, 1998). This initiated from the apex, with the remainder of the NST becoming progressively purple during dehydration (**Figure 2C**). Upon rehydration, the leaf blades of NST unfolded progressively as water moved from the base toward the apex. However, a large portion of the apical tip tissues did not unfold, this correlating with the low RWC of the ST (<20% RWC, **Figure 2D**). NST were yellow in color during initial rehydration, regaining their green color by 72 h post-watering, this correlating with a 30% increase in chlorophyll content (**Supplementary Figure 1**). Interestingly, the zone between NST and ST tissues became demarcated by a purple band that remained permanently visible after rehydration, this tissue being designated for senescence on the next desiccation cycle (**Figure 2D** inset).

**Figure 3:**
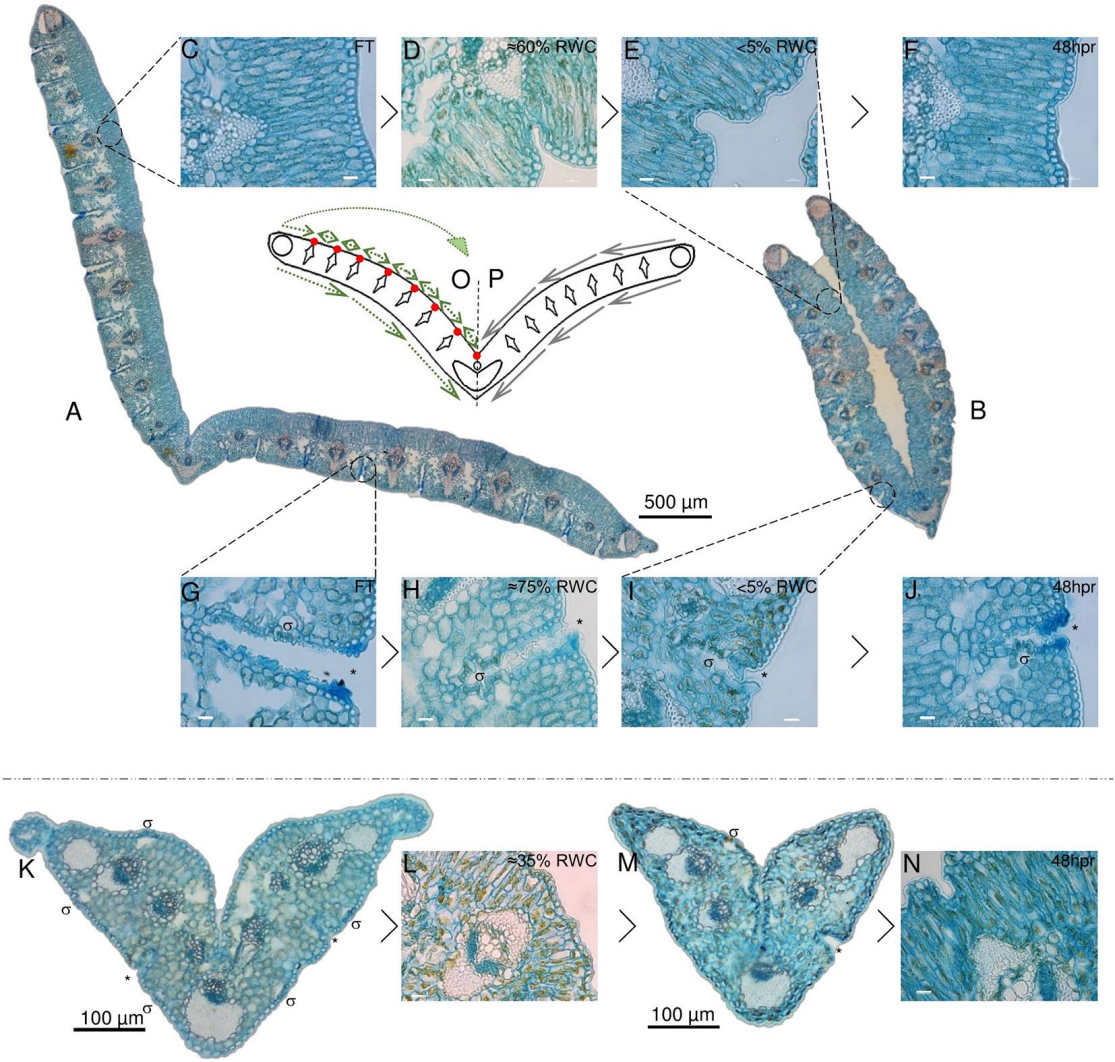
Desiccation associated tissue modifications in non-senescent tissues (NST) and ST. Typical cross section of a full turgor [≈100% relative water content (RWC); FT] blade is shown in **(A)** with while a cross section at 5% RWC is shown in **(B)**. The abaxial surface in A is open to the environment, while this same surface becomes enclosed due to folding about the midrib in **(B)**. Modifications to the adaxial palisade mesophyll during desiccation and at 48 h post-rehydration (48 hpr) is shown in **(C**–**F)** with %RWC indicated. Modifications to abaxial stomatal channels is shown in **(G**–**J)**. Stomata (σ) lay in deep channels (*) **(G**–**J)** that become increasingly closed and mucilage-rich **(H**, **I)** with water deficit stress. ST is shown in **(K**–**N)**. Stomatal channels appear not to close as efficiently **(M)** and there appears to be a greater degree of Procion yellow staining, indicating a higher degree of cellular constituent aggregation. **(O**, **P)** represent a simplified model of the typical leaf blade in cross section with all collections of sclerenchyma indicated. **(P)** displays the theoretical mechanical forces experienced by the tissues at water loss, while **(O)** displays a hypothesized means by which points of palisade compaction allow for redirection of these forces to provide the observed transition from **(A**–**B)**. Scale bars for **(C**–**J**, **L**, and **N)** are 10 µm, rest are as indicated.

The anatomical changes associated with dehydration and recovery of NST and ST of *X. schlechteri* are shown in **Figure 3**. Details of leaf anatomy and changes therein during desiccation of *X. schlechteri* have not been previously described. Transverse sections of fully hydrated leaves show a regular modular arrangement, each consisting of a central *Vellozia*-type vascular bundle (Behnke et al., 2013), flanked by deep abaxial channels that extend through the majority of the spongy mesophyll (**Figures 3A, B**). Most of the abaxial stomata occur within these channels, interspersed by epidermal cells (**Figures 2G–J**). Vascular bundles are capped both ad- and abaxially by sclerenchyma, which extend into the adaxial palisade mesophyll (**Figure 3C**). Three large bundles of sclerenchyma fibers are present, one at each lateral edge of the blade and one at the midrib (**Figures 3A, B**). These run in parallel from the base of the blade to the apex and likely provide uncompressible points during desiccation.

**Figure 3A** depicts a cross section of fully hydrated NST. Upon dehydration of this tissue, the palisade mesophyll cells became increasingly compacted, causing infolding of the adaxial epidermis adjacent to the vascular bundle of each module (**Figures 3D, E**). These points of compaction possibly allow for the redirection of the mechanical forces associated with water loss (Iljin, 1957) and ultimately the folding of the leaf blade (**Figure 3B**). A model depicting these mechanical forces is given in **Figures 3 O, P**, with P depicting the proposed forces experienced by the leaf blade during desiccation and O representing the hypothesized means by which the points of compaction alter the direction of these forces, such that the resultant leaf folding is produced in the desiccated NST (**Figure 3**). At the abaxial surface, a reduction in mesophyll cell size was observed as is typical of dehydrating tissue, but the most striking observation was the apparent subcuticular excretion from the epidermal cells of acid complex polysaccharides (as evidenced by Alcian blue staining; Parker & Diboll, 1966), this possibly being mucilage, into channels containing stomata (**Figure 3H**). These subcuticular secretions increased on drying (**Figure 3I**), and we postulate that the presence of mucilage might retard the rate of water loss from mesophyll cells and ultimately contribute to the timing of stomatal closure. Upon rehydration, the palisade cells expanded and epidermal infoldings were no longer evident by 48 h (>75%), this corresponding with observable unfolding of the leaf blade. At this stage, some secretions were still evident in the abaxial channels (**Figure 3J**), but these had begun to resemble the FT tissue by 72 h post-rehydration.

At full hydration, ST have a RWC of *ca*. 65%, and the appearance of the leaf tissues (**Figure 3K**) was not entirely dissimilar to NST at similar water contents (**Figures 3D, H**). While there was little evidence of adaxial epidermal folding, abaxial stomatal-containing channels were positively stained by Alcian blue (asterisk in **Figure 3K**). Further dehydration, however, appeared to result in less ordered structural changes than were evident in NST at similar water contents. Palisade cells appeared warped (**Figure 3L**), and some stomatal channels appeared to have been forced open probably due to the strain associated with desiccation and incomplete plugging of channels by mucilaginous substances (**Figure 3M**). The ST did not recover structurally during rehydration and resembled the air-dry state at 48 h post-rehydration (**Figure 3N**).

**Figure 4:**
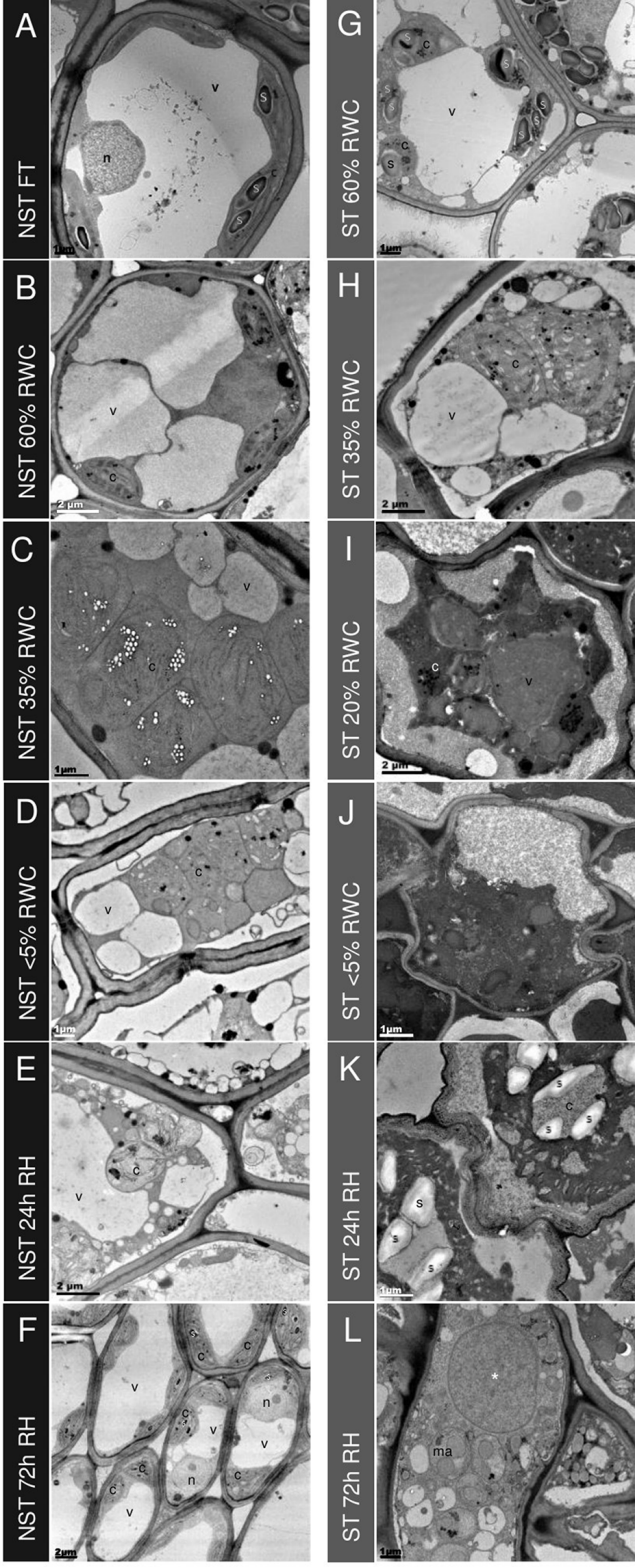
Ultrastructural changes during desiccation and rehydration. Transmission electron micrographs of mesophyll cells in non-senescent tissues (NST) (left column A-F) and ST (right column G-L) from the same leaves during desiccation and rehydration, with relative water content (RWC) for each on the left. When NST mesophyll cells are at FT **(A)**, a central electron opaque vacuole is surrounded by active (indicated by the presence of starch) chloroplasts with good thylakoid organization. As plants experience water deficit, the central vacuole splits into several smaller vacuoles **(B**–**D)** and the chloroplasts become swollen and lose thylakoid membrane organization. Once rehydrated **(E**–**F)**, NST mesophyll quickly regains the structure observed in the hydrated state, with reformation of the central vacuole observed at 24 h **(E)** and thylakoid organization regained by 72 h **(F)**. The presence of starch granules in peripheral chloroplasts indicates regaining of photosynthetic competence **(F)**. Mesophyll cells of the ST **(G**–**L)** experience lower corresponding water contents during water deficit and this is evident on the ultrastructural level with mesophyll cells from fully hydrated plants (60% RWC in ST) resembling the organization of drying NST (G compared to B). Drying below 35% in ST results in a different appearance of mesophyll in ST from that of NST—the cytoplasm becomes increasingly electron dense **(I**–**L)** and there appears to be contraction of the membrane from the cell wall **(I**–**J)**, and cell wall folding **(J**–**K)**. Of the cells that are not lysed in rehydrated mesophyll **(L)**, there is evidence of macroautophagy and the appearance of a large electron dense vesicle (*). Symbols are used to indicate organelles: vacuoles (v), nuclei (n), chloroplasts (c), starch granules (s), electron dense vesicle (*), and macroautophagosomes (ma).

The ultrastructural changes of the mesophyll cells of NST and ST during dehydration and recovery are shown in **Figure 4**. The changes in subcellular organization observed in NST were typical of that reported for this and other desiccation tolerant *Xerophyta* spp. (Sherwin and Farrant, 1998; Farrant, 2000; Farrant et al., 2007), characterized by increased presence of smaller vacuoles, centralization of chloroplasts and progressive dismantling of thylakoid membranes, and increased presence of plastoglobuli during dehydration (**Figures 4A–D**). In resurrection plants, mechanical stabilization is achieved through a combination of cell wall flexibility and the presence of vacuoles containing non-aqueous metabolites, with species exhibiting less cell wall flexibility having a greater degree of vacuolation (Farrant et al., 2007; Farrant et al., 2012). The vacuolar content appeared to be uniformly electron dense during dehydration (**Figure 4D**). In the present study, thylakoid organization was still evident at 55% RWC, but starch was no longer present (**Figure 4B**) and complete dismantling of the thylakoid membranes was evident by 35% RWC (**Figure 4C**). In parallel with thylakoid dismantling, there was a significant decline in chlorophyll content, with losses of 80% by 55% RWC and near complete loss in the air dry state (**Supplementary Figure 1**), confirming the poikilochlorophyllous nature of *X. schlechteri*.

The STs exhibited a lower water content than their NST counterparts at any one time point, but interestingly, the general appearance of subcellular organization was initially not dissimilar to that of NST at similar water contents (compare G with A and H with C in **Figure 4**). Photosynthetic pigments were also consistently lower in ST but were not dissimilar to those of NST at similar water contents (**Supplementary Figure 1**). This might suggest that the changes observed are associated with water content, rather than the engagement of different metabolic strategies between the two tissue types. At RWCs lower than 35%, however, the subcellular organization of ST differed from that of NST. Considerable plasmalemma withdrawal from the cell wall was evident and the extra cytoplasmic space between the wall and plasmalemma was electron dense, suggesting the presence of osmophilic substances (**Figures 4I, J**). While some plasmalemma withdrawal might be an artifact of the method of chemical fixation used [this being evident also in NST (**Figure 4D**)], the extent of withdrawal might be related to insufficient contribution of vacuolar area to mechanical stabilization. In most cells, vacuole-like organelles containing uniformly electron-dense material were observed (see * in **Figures 4I, L**), but the extent of vacuolation was not as great as in NST at similar water contents. This suggests a completely different vacuolar content between the two tissue types. Chloroplasts were evidenced predominantly by the presence of plastoglobuli (**Figures 4I, J**).

Twenty-four hours after soil watering, the water content of NST was 60% RWC, while that of ST remained at < 5%. In NST, mesophyll cells had largely regained the central vacuole, and membranous strands resembling early thylakoids were present in the chloroplasts (**Figure 4**). In contrast, ST remained similar in appearance to that of the desiccated state, with the exception of the presence of starch in what can be assumed to be ex-chloroplasts, as it is unlikely that any organelle biogenesis had occurred during the 24 h period since soil watering.

By 72 h post-hydration, the mesophyll cells of NST (**Figure 4F**) were similar to those of fully hydrated tissue prior to dehydration (**Figure 4A**). Chloroplasts had well developed thylakoid membranes, chlorophyll content had increased (**Supplementary Figure 1**), and starch was present, suggesting photosynthetic competence. In ST, now at 20% RWC, many mesophyll cells had lysed (**Supplementary Figure 7A**), while some appeared to have limited organization (**Figure 3L**, **Supplementary Figures 7B–F**). Starch was no longer evident in plastids, indicating potential operation of amylases. Electron dense vacuole-like organelles were still evident (indicated by *), but several smaller macroautophagic-like vacuoles, containing remnants of organelles and cytoplasmic debris, were also discernible, suggesting that autolysis might be occurring in these tissues after rehydration.

### Changes in Primary Metabolites With Dehydration

**Figure 5:**
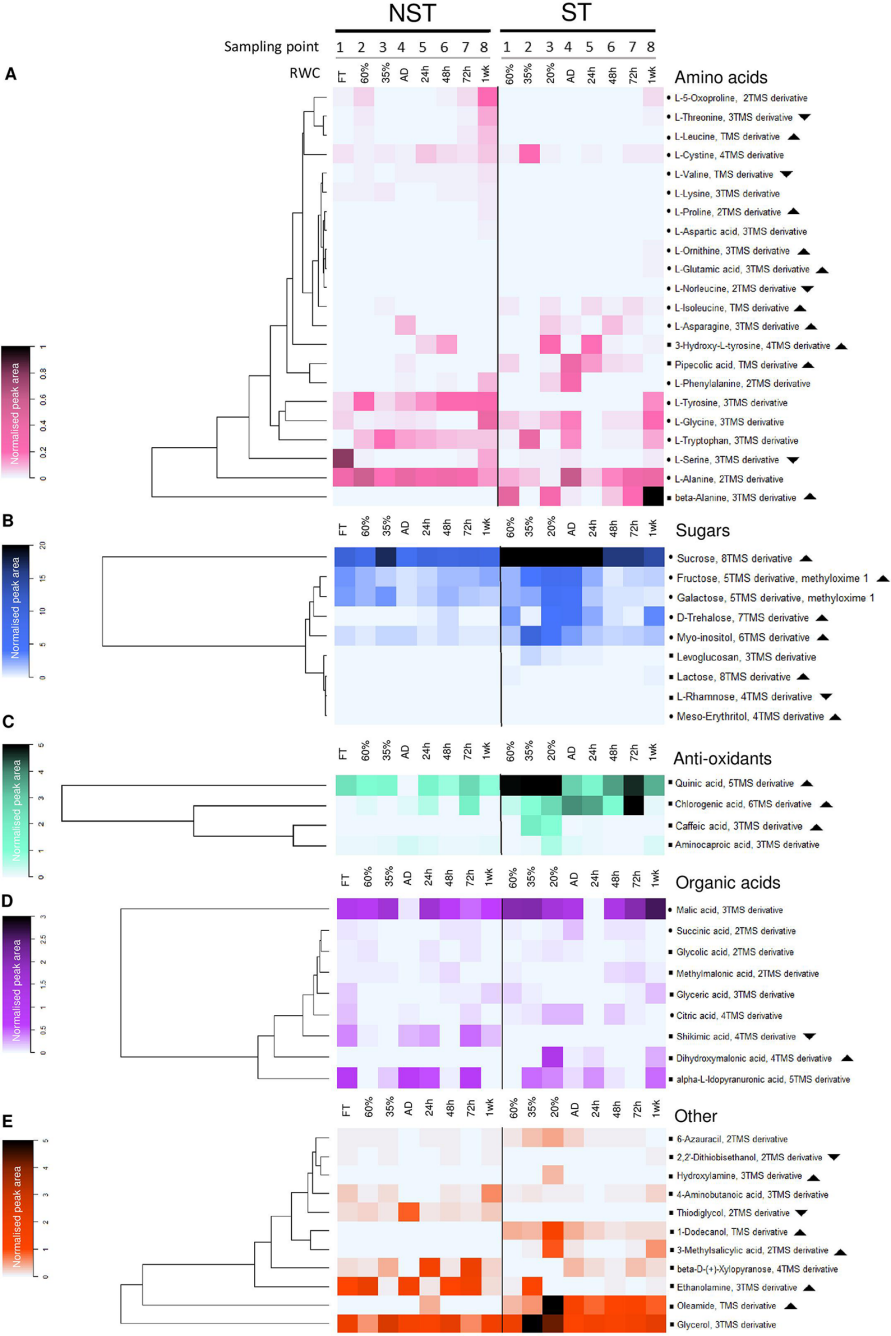
Desiccation associated responses in the metabolome of non-senescent tissues (NST) and ST as determined by gas chromatography–mass spectrometry. Average peak area of compounds identified in this study, normalized to ribitol internal standard and sample dry masses. Note that for each sampling point (1-8), ST and NST from the same leaves differ in average RWC. In each of the compound classes is a dominant (highly accumulated) metabolite: alanine **(A)**, sucrose **(B)**, quinic acid **(C)**, malic acid **(D)**, and glycerol **(E)**. Symbols: indicates compound identification was done using the National Institute of Standards and Technology (NIST) library and an internal library of standards; indicates NIST library identification only; indicates significantly greater total accumulation in ST *vs*. NST (log_2_ fold change ≥2, t-test p ≤ 0.05, FDR ≤ 0.1); indicates significantly lesser total accumulation in ST *vs*. NST. AD refers to the airdry state.

Previous molecular physiological studies on *X. schlechteri* have demonstrated that key changes associated with desiccation tolerance are initiated at RWCs below 55%, this correlating with the shutdown of photosynthetic carbon gain (Mundree and Farrant, 2000; Farrant et al., 2015). Dehydration below *ca*. 40% leads to further significant changes (both increased and decreased) in transcript abundance, with the tissues appearing to enter a quiescent state below 10% RWC (Costa et al., 2017). Similar trends were observed in the suites of primary metabolites examined in the present study, in which changes in the concentrations of many metabolites were evident below 60% RWC, with further shifts being evident at 35% RWC and the air-dry state (**Figure 5A**, **Supplementary Figures 2–6**). If we assume that photosynthesis was shut down below 55% RWC, as suggested in previous studies and corroborated by the ultrastructural observations in the present study (**Figure 4**), then the nature of the metabolites present is likely to be reflective of the native metabolism in this species and shifts in concentrations during drying to 60% RWC are probably related to osmoprotection and the prevention of oxidative stress. The accumulation of amino acids, including threonine, alanine, tryptophan, 5-oxoproline, tyrosine, and leucine (**Figure 5**, **Supplementary Figure 2**), was observed at this stage. Other amino acids remained similar or declined in abundance at this water content. Among the sugars and sugar alcohols examined, there was an increase in trehalose and rhamnose and a slight decline in myo-inositol, fructose, galactose, β-D-xylopyranose, lactose, and glucose (for glucose, see **Supplementary Figure 1**). Sucrose concentrations also diminished slightly, but were overall consistently high, being the dominant metabolite with a peak area 3 or 4 orders of magnitude greater than the other metabolites in all samples. The antioxidant chlorogenic acid, as well as organic acids, glycolic acid, and methylmalonic acid increased in concentration during dehydration to 60% RWC. Among the TCA cycle intermediates, all except succinic acid declined during drying to this water content, suggesting a slowing-down of respiration.

Dehydration to 35% RWC resulted in a shift from source tissue metabolism to protective metabolism in NST, as carbon assimilation could no longer take place. This shift resulted in the maintenance or decline in abundance of most metabolites tested, with the exception of glutamic acid, ornithine, lysine, tryptophan, fructose, galactose, levoglucosan, citric acid, and 1-dodecanol, which increased in abundance (**Figure 5**, **Supplementary Figures 2**–**6**). On further dehydration toward the air-dry state, there were increases in concentrations of the amino acids asparagine, aspartic acid, phenylalanine, ornithine, glutamic acid, alanine, tryptophan, tyrosine and pipecolic acid, the organic acids citric acid, methylmalonic acid and aminocaproic acid, and hydroxylamine.

As outlined above, ST was maintained at lower water contents than NST on the same leaves. However, metabolism did not resemble that of the NST tissues at similar RWCs, and the metabolite trends between 60% and AD were more pronounced than in the NST. Amino acid accumulation is predominantly where NST and ST deviated in terms of metabolic profile: ornithine, proline, β-alanine, norleucine, pipecolic acid, serine, 3-hydroxy-L-tyrosine, aspartic acid, and glutamic acid were present in higher amounts in ST throughout dehydration and desiccation. As observed in NST, ST significantly accumulated phenylalanine and asparagine in the dry state. Unlike in NST, serine, glycine, ornithine, β-alanine, and pipecolic acid accumulated significantly (**Figure 5**, **Supplementary Figures 2** and **3**). The sugars trehalose, sucrose, fructose, levoglucosan, lactose, and myo-inositol remained consistently elevated in ST, while glucose followed similar declines to NST (**Figure 5**, **Supplementary Figures 1**–**4**). Similarly, caffeic and quinic acid contents remained elevated in ST relative to NST throughout drying (**Figure 5**, **Supplementary Figure 5**). In the desiccated state, citric acid levels were elevated to the same degree as in NST, but unlike in NST, succinic acid was elevated rather than diminished. Most other organic acids were unchanged or diminished (**Figure 5**, **Supplementary Figure 5**).

### Changes in Primary Metabolites During Rehydration

Most studies on resurrection plants have measured the metabolic changes occurring upon rehydration to full turgor and initiation of photosynthetic competence, with the longer-term metabolic changes being omitted. In such studies, early rehydration is characterized by an increase in TCA metabolites, correlating with rapid increases in respiration. Levels of protective sugars (sucrose, raffinose, stachyose, and verbascose) decline, whereas antioxidants (superoxide dismutase, glutathione reductase, quinic acid), osmoprotectants, and N storage metabolites (various amino and organic acids) tend to remain elevated (Moyankova et al., 2014; Suguiyama et al., 2014; Yobi et al., 2017). In this study, while full photosynthetic competence is reported to occur by 72 h of rehydration (Farrant et al., 2015), tissues were also sampled at 1-week post-watering when the NST of all plants sampled had reached full turgor (**Figure 2**).

After 24 h of rehydration (average tissue RWC of 50%), there was a return to basal (pre-dehydration) levels of citric acid and an increase in malic acid, as well as declines in sucrose, glucose, and galactose in NST, which suggests the early resumption of respiration. Increased levels of quinic and chlorogenic acids imply that additional antioxidant protection was required at this stage (**Figure 5C**). There was also an increase in trehalose content, which remained elevated until 48 h after rehydration. By 72 h, except for the above-mentioned antioxidants and the amino acids tryptophan and tyrosine, which remained elevated relative to the hydrated state, other metabolites had returned to basal pre-dehydration levels. Increased levels of shikimic acid might imply that metabolism *via* the shikimate pathway was occurring, corroborated by the elevation of tryptophan and phenylalanine at 1 week post-rehydration. At this stage, tyrosine remained elevated and there were increased levels of threonine, cysteine, glycine, and serine relative to the pre-desiccated state.

Whereas the NST reached full turgor within 72 h, the ST was maintained at ∼20% RWC. Despite this, there was evidence of metabolic activity in these tissues, with many of the metabolites indicating similar temporal trends as the NST (**Figure 5**, **Supplementary Figures 2** and **3**). After an initial decline at 24 h, the amino acids asparagine, glycine, serine, β-alanine, and alanine accumulated toward the later stages of rehydration. Some amino acids followed similar trends to those in NST (5-oxoproline, leucine, phenylalanine, threonine) (**Figure 5**, **Supplementary Figures 2** and **3**). The sugars trehalose, levoglucosan, sucrose, and myo-inositol were consistently elevated in ST but tended to follow a similar pattern to NST during rehydration (**Figure 5**, **Supplementary Figure 4**). Citric and succinic acid very closely mimicked the accumulation patterns in their NST counterparts, except for accumulation peaking at 20% RWC. Shikimic acid peaked significantly at 72 h and remained high after 1 week of rehydration, corresponding with increases in tryptophan, tyrosine, 3-hydroxytyrosine, and phenylalanine abundance, indicating that the shikimate pathway was active 1 week after rehydration (**Figure 5**, **Supplementary Figures 2**, **3**, and **5**).

### Changes in Other Compounds

The anoxic nitrate metabolite hydroxylamine (Sturms et al., 2011), present in desiccated NST and at 24 h post-rehydration, diminished significantly at 48 h (**Figure 5E**), indicating a return to oxygenated conditions after anoxic quiescence. This compound remained elevated in ST throughout dehydration and rehydration, suggesting that the ST remains anoxic until 1 week following rehydration. The fatty acid derivative oleamide and fatty alcohol 1-dodecanol presumably appear in this data set as they were sufficiently polar to be extracted in the methanol fraction. These compounds, after peaking in NST at 24 h post-rehydration and the desiccated state respectively, returned to basal levels after 48 h rehydration. Both were elevated in ST relative to NST at all times (**Figure 5**, **Supplementary Figure 6**). The sulfuric compounds thiodiglycol and 2’2-dithiobisethanol, while present in the NST, did not fluctuate much during dehydration and rehydration and were detected at very low levels in ST (**Figure 5**, **Supplementary Figure 6**). The cell wall components β-D-xylopyranose (Zhou et al., 2018) and levoglucosan were elevated at 35% RWC in NST, corresponding with increases in other sugars at this stage.

## Discussion

Desiccation tolerance is a complex multigenic trait and is achieved *via* an intricate suite of molecular, biochemical, and physiological mechanisms geared toward mechanical stabilization, mitigation of photo-oxidative damage, stabilization of key proteins and enzymes, and repair upon rehydration (Farrant et al.,2007; Gechev et al., 2012; Moore et al., 2013; Karbaschi et al., 2015; Challabathula et al., 2016; Blomstedt et al., 2018).While much work has been done on the molecular biology of this species (Costa et al., 2017; Artur et al., 2019), little work has been done on understanding its anatomical and metabolic adaptations for desiccation tolerance. Understanding these in surviving tissues, in comparison with metabolic changes in apical tissues which undergo senescence on desiccation, gives insight as to how senescence is repressed in the former yet proceeds in the latter, and provides a foundation for future work. Here we describe the drying and rehydration of NST, combining observations from this study and previous studies on whole leaves from *X. schlechteri* and other resurrection plants. We hypothesize on how senescence is initiated in response to aging and water deficit and describe the progression of this process during and following desiccation.

### Mitigation of Mechanical, Osmotic, and Photo-Oxidative Stress During Dehydration in Non-Senescent Tissues

At full turgor, leaf anatomy and mesophyll ultrastructure are indicative of metabolically active tissues. Stomatal channels are open and free of mucilage, suggesting active photosynthesis and gas exchange. Sugars, organic acids, and TCA cycle intermediates are highly accumulated. Sucrose, alanine, quinic acid, and glycerol levels are high, and remain so during desiccation and rehydration, suggesting that they play a role as constitutive ameliorators of abiotic stressors associated with water deficit.

Dehydration to 60% RWC results in initiation of changes that mitigate against mechanical, osmotic, and photo-oxidative stresses. Infolding of tissues adjacent to vascular bundles at this RWC allows for the redirection of mechanical forces associated with water loss (Iljin, 1957), likely alleviating much of the mechanical stress, while allowing for folding of the whole leaf at the midrib. The observed subcuticular excretion of mucilage by epidermal cells into abaxial stomatal channels serves to force the associated cuticle into close proximity, closing the stomatal channel aperture and limiting the rate of water loss while potentially still allowing some gas exchange. At the subcellular level, mechanical stabilization is achieved by division of the central vacuole into several smaller vacuoles which collectively maintain subcellular volume, preventing plasmalemma detachment from the cell wall, as has been reported for resurrection plants which have less flexible cell walls (Farrant et al., 2007; Farrant et al., 2012). Metabolically, the accumulation of osmolytes such as sugars and amino acids have been proposed to slow water loss and maintain cell turgor (Fàbregas and Fernie, 2019). Oliver et al. (2011) have ascribed the accumulation of amino acids at 60% RWC in the resurrection grass *Sporobolus stapfianus* to affording such osmoprotection, and we propose that the increase in the amino acids threonine, alanine, tryptophan, 5-oxoproline, tyrosine, proline, and β-alanine together with consistently elevated sucrose levels in *X. schlechteri* also serve this purpose. At this point, chloroplasts still have stacked grana, but there is little/no starch and chlorophyll content was a third of that in leaves at full turgor. These observations, coupled with the relative diminishment of fructose, glucose, and TCA intermediates, indicate slowing of photosynthesis and carbon gain, corroborating previous studies on this species (Mundree and Farrant, 2000; Farrant et al., 2015). Such studies have also shown increased activities of several antioxidant enzymes, proposed to play a role *inter alia* in protection against photo-oxidative stress during early dehydration. The present study demonstrates that the antioxidant quinic acid is constitutively high in NST and is accompanied by the accumulation of chlorogenic acid, these possibly adding to photo-protection required on drying to this water content. The increase in trehalose content at 60% RWC could explain maintained sucrose levels observed in this species. Sucrose regulation has been proposed to occur *via* the interaction of trehalose-6-phosphate (Tre6P) and Suc-non-fermenting-1-related kinase 1 (SnRK1), termed the sucrose Tre6P (Suc-Tre6P) nexus, in which Tre6P acts as both a signal and a negative feedback regulator of sucrose levels in plants, maintaining optimal levels under all conditions (Zhang et al., 2009; Yadav et al., 2014; Oszvald et al., 2018). Since Tre6P is essential to trehalose formation, it is possible that its presence did allow interaction with SnRK1, so enabling high levels of sucrose after cessation of photosynthesis. While operation of this regulatory pathway has been postulated in resurrection plants (Blomstedt et al., 2018) this has not yet been clearly demonstrated.

Dehydration to 35% RWC results in complete shutdown of photosynthesis as evidenced by thylakoid dismantling and complete loss of chlorophyll. Declines in fructose, glucose and shikimic, citric, and succinic acid indicate a decline in glycolysis and the TCA cycle and a potential carbon shift toward protection. At the cellular level, mechanical stabilization is afforded by increased vacuolation and chloroplast swelling and is inextricably linked with metabolism. It has been proposed that in desiccation tolerant organisms water is progressively replaced with vitrifying sugar solutions (Hoekstra et al., 2001; Buitink and Leprince 2004; Farrant et al., 2012). In *X. schlechteri*, peak concentrations of sucrose, glycerol, and malic acid are present at this water content. Sucrose content is constitutively high in this species, exhibiting only a two-fold increase during drying, unlike most other resurrection plants reported on which sucrose content elevates more dramatically during desiccation (Bianchi et al., 1991; Farrant et al., 2007; Peters et al., 2007; Oliver et al., 2011; Mladenov et al., 2015). It is likely that this sugar is the principal vitrifying agent in this species. However, it is also possible that it participates in the formation of natural deep euctectic solvents (NaDEs), which have been proposed to form a third liquid phase (other than water or lipid) in which cellular components can be effectively concentrated and proteins protected from denaturation (Choi et al., 2011). Malic acid, sucrose, and glycerol form a NaDES in a 1:1:2 ratio (Dai et al., 2013) and could be contributing toward subcellular stabilization and potentially facilitate ongoing metabolism reported to occur at low water contents in this species (Mundree and Farrant, 2000; Farrant et al., 2015; Costa et al., 2017). Interestingly, β-D-xylopyranose and levoglucosan (a cell wall sugar) peak at 35% RWC suggesting metabolic activity within cell walls. The reason for this accumulation is unclear but could reflect marginal changes in wall architecture (Moore et al., 2013).

Upon further dehydration to the air-dry state, desiccation tolerant tissues are proposed to become quiescent and/or dormant (Farrant et al., 2012; Gechev et al., 2012; Costa et al., 2016). In *X. schlechteri* metabolic quiescence is entered with a decline in malic acid and an accumulation of citrate, reflecting shut down of respiration, reported to occur at this stage (Mundree and Farrant, 2000). The accumulation of hydroxylamine, an anoxic nitrate metabolite (Sturms et al., 2011), indicates that there is some degree of anoxia following respiratory shutdown. Interestingly, there was a notable accumulation of the amino acids asparagine (its only peak in accumulation), aspartic acid, phenylalanine, ornithine, glutamic acid, alanine, tryptophan, and tyrosine in the dry state which we propose could serve as nitrogen storage for rapid remobilization and reincorporation into proteins upon rehydration, and/or as osmoprotectants (Djilianov et al., 2005; Oliver et al., 2011).

### Recovery of Non-Senescent Tissues

Recovery to full turgor occurs only after 72 h, yet considerable structural and metabolic changes occur prior to this. By 48 h after receipt of water, infolding of palisade cells was relieved and both mesophyll and palisade cells take on the shape observed in fully hydrated tissues. At this stage, chloroplasts regain some thylakoid organization, but chlorophyll levels remain low and it is likely that they only gain photosynthetic competence at and after 72 h, where starch formation begins again. This correlates with previous reports on recovery of photosynthetic competence in this species (Sherwin and Farrant, 1996). Thus, the metabolites that were accumulated during desiccation are crucial for metabolic activity in the early stages of rehydration, where minimal photosynthetic activity drives the synthesis of ATP. We propose that sucrose acts as a major carbon source for the biosynthesis of cellular constituents and regeneration of ATP *via* glycolysis during early rehydration. Similarly, asparagine, which has been proposed to be the major nitrogen storage compound in *S. stapfianus* (Oliver et al., 2011), diminishes considerably upon rehydration and thus, like sucrose, is probably utilized for the generation of other necessary metabolites. Poikolochlorophyllous resurrection plants are sensitive to photo-oxidative stress during restoration of photosynthetic competence (Sherwin and Farrant, 1996; Tuba et al., 1998) and elevated levels of carotenoids and the antioxidants chlorogenic and quinic acid, which peak at 72 h RH are likely to contribute to photoprotection. By 72 h, recovery of shikimic acid and the biosynthetic end-points phenylalanine, tyrosine, and tryptophan is observed. The same is true for TCA intermediates, implying that energy production and biosynthesis proceeds *via* these pathways, fed partially by sucrose and asparagine and partially by photosynthesis. The significant increase in trehalose during the first 48 h could again suggest operation of the Suc-Tre6P nexus, this possibly regulating sucrose content until photosynthetic competence is gained. Alternatively, it is possible that here trehalose is playing a role in autophagy, allowing clean up of subcellular damage or macromolecular aggregates that may have formed during desiccation and quiescence (Williams et al., 2015; Asami et al., 2019).

Understanding the fundamental mechanisms underlying desiccation tolerance in this species enables us to study senescence with a clearer lens. Is senescence simply a system failure in the oldest tissues, wherein they succumb to the stresses imposed by desiccation? Is it initiated and conducted in a controlled manner? Are age-related senescence processes exacerbated by drying?

### Dissecting the Lower Relative Water Content Phenomenon and Progression of Senescence in Senescent Tissues: Anatomical and Metabolic Arguments

There are three broad processes that characterize leaf senescence: initiation, reorganization, and termination (Bresson et al., 2018). Without application of external stress, leaf age and developmental stage are the main initiators of senescence. Early onset of senescence can also be triggered by abiotic and biotic stressors, driven by hormones, sugars, and ROS generated by these processes. The reorganization phase is characterized by the breakdown of macromolecules and remobilization of nutrients in small subunit form, wherein hydrolytic enzymes are extensively deployed (Bresson et al., 2018). Basic metabolic activity during this phase is required to ensure remobilization of breakdown products to the vasculature for incorporation in viable cells. Toxic intermediates are likely to form during this phase, and thus antioxidant enzymes and compounds (particularly anthocyanins) are deployed to protect against oxidative stress in this state (Bresson et al., 2018). During termination, vacuoles collapse, releasing nucleases and proteases into the cytoplasm, leading to acidification and contraction of the cytoplasm and uninhibited breakdown of DNA, RNA, proteins, and membranes. At this point, the cell is effectively dead, leaving behind cell wall and debris (Lim et al., 2007; Bresson et al., 2018).

Tracking senescence is challenging in poikilochlorophyllous resurrection plants, which employ many of the “reorganization” mechanisms as part of their desiccation tolerance programming (chlorophyll degradation, lipid changes, metabolic shifts, accumulation of compatible solutes, increased abundance of chaperones, antioxidant accumulation, etc.) (Oliver et al., 2011; Gechev et al., 2012; Christ et al., 2014; Farrant et al., 2015). Comparison of anatomy, ultrastructure, and metabolism of ST to that of NST is the logical first port of call in the pursuit of understanding senescence processes in leaves. To begin with, under well-watered conditions, ST is maintained at a lower water content (1.8–2.2 g/g dry mass) than NST (2.8–3 g/g dry mass). There are two potential explanations. Firstly, it is possible that these tissues have a lower osmotic potential and lack of active transpiration might slow the diving force of water, leaving the cells with a lower water status. Ultrastructural studies show limited thylakoid organization in the fully hydrated state, confirming limited photosynthetic activity driving water potential. A second likely explanation for this phenomenon is that the water content of cells within these tissues is not homogenous. The termination phase of senescence is associated with lysis of the vacuole and the consequent breakdown of cellular structure and plasmalemma rupture. When water content determination is performed, it is done so on a dry mass basis. Lysed cells are likely to hold less water as there are no intact membranes to contain it, but cell walls contribute toward total mass of the tissue. Thus, the impression could be made that tissues are held at an overall lower RWC, where this is true of only some cells. In the present study however, while subcellular organization of most mesophyll cells of ST at RWC below 35% was compromised, there was little evidence of complete subcellular lysis until rehydration. Thus this hypothesis holds when considering the water contents of the ST mesophyll cells after 48 and 72 h rehydration.

Ultrastructurally and metabolically, there is evidence that age-related senescence processes are ongoing in well watered plants and are exacerbated during desiccation. In fully hydrated plants, ST has an average RWC of ∼60%. Chloroplasts contain significantly more starch than NST and less chlorophyll and carotenoids at similar water contents, typical of early stage age-related senescence in *Arabidopsis thaliana* (Watanabe et al., 2013). Other similarities to *A. thaliana* age-related senescence in ST at this water content are the relatively high levels of TCA intermediates, glycerol, trehalose, the antioxidants quinic and chlorogenic acid and persistent presence of anthocyanins in ST (Watanabe et al., 2013). Furthermore, in terms of amino acids, β-alanine glycine, pipecolic acid, and isoleucine are elevated in ST whereas threonine, alanine, tryptophan, 5-oxoproline, tyrosine, and proline are not, suggesting the NST is geared toward osmoprotection and ST toward the initiation of senescence.

Upon dehydration to ∼35% RWC, ST displays further evidence of ongoing senescence processes. Mesophyll and palisade cells exhibit a high degree of Procion yellow staining compared to NST at similar and lower RWCs, indicating stress and aggregation of cellular constituents. While ultrastructurally, most cells of ST appear similar to that of NST at 35% RWC, metabolically these tissues have different signatures. In ST fructose, galactose, and myo-inositol are elevated and additional antioxidant protection is provided in the form of elevated chlorogenic and quinic acid, and of caffeic acid, which appears to be exclusively elevated in ST. These responses closely resemble the “reprogramming” phase of senescence as described in *A. thaliana* (Watanabe et al., 2013), with the exception of vacuolation, which is a desiccation tolerance phenomenon (Farrant et al., 2015).

Dehydration to the air-dry state results in further significant differences. In the ST, the cytoplasm is electron-dense and the plasmalemma appears withdrawn from the cell wall, indicating severe osmotic stress. Chloroplasts are discernible only by the presence of plastoglobuli and appear shrunken. There is significant cell-wall folding, indicating either deliberate remodeling of cell walls in an attempt to mitigate the mechanical stress, or cell wall collapse and redistribution of non-structural sugars. Metabolically, this is corroborated by the elevation of levoglucosan exclusively in the ST during desiccation, starting at 35% RWC. Methyl-salicylic acid content also peaks at this point and has been shown to positively regulate leaf senescence in water-stressed plants (Abreu and Munné-Bosch, 2008) and protect against fungal infection (Dieryckx et al., 2015), suggesting that a potential signaling role is played here. While there was some metabolic overlap with NST at low water contents, persistent differences were the higher accumulation of fructose, galactose, trehalose, alanine, glycine, pipecolic acid, and phenylalanine, and a relative lack of asparagine and tyrosine.

Upon rewatering there is little increase in water content until 48 h, when ST reach 10% RWC. Varying degrees of subcellular disorganization is evident with some cells being relatively intact and resembling the air-dry state, with others being entirely lysed. This pattern is maintained upon further rehydration to the ultimate maximum value of 20% RWC at 72 h post-watering and is typical of the termination phase of senescence (Bresson et al., 2018). Interestingly, cells that maintain subcellular organization have electron dense vacuole-like structures and there is evidence of macroautophagy. This differential pattern of cellular integrity suggests different levels of hydration could be occurring among cells in ST and supports the second hypothesis outlined above in accounting for differential water contents in NST and ST. There was also no evidence of recovery of photosynthetic competence nor operation of the shikimic acid pathway observed in NST.

## Conclusion

*X. schlechteri* undertakes a variety of anatomical, ultrastructural, and metabolic responses to desiccation, responding to the unique requirements associated with each phase of water loss and recovery. Similarities between the reorganization phase in senescence and desiccation-associated changes make studying senescence in resurrection plants difficult. The “tip-burning” phenomenon in this species enables the study of viable and ST from the same leaves, and so enabled comparison in this study. Both tissues executed their desiccation tolerance programming: both limited the rate of water loss through accumulation of mucilage in stomatal channels, both reorganized chloroplasts and formed numerous vacuoles, both accumulated protective metabolites. Drying below 35% RWC in ST resulted in exacerbation of age-related senescence processes already underway in these tissues, resulting in damage to and potential loss of many cells in these tissues on desiccation. This in turn is exacerbated on some hydration but prevents ultimate rehydration of these tissues to levels observed before water deficit stress. Further study in this area will be aimed at understanding how senescence processes are transcriptionally regulated in this species, looking at cross talk between age-related and dehydration-induced transcriptional regulation.

## Data Availability Statement

All datasets generated for this study are included in the article/**Supplementary Material**.

## Author Contributions

All authors contributed toward the design of the experiments and interpretation of results. ST conducted light microscopy and colorimetric assays and AR conducted the transmission electron microscopy and GC-MS metabolite aspects of the study. JMF supervised the project. The manuscript was written by AR, with all authors contributing to and revising thereof.

## Funding

The work was funded by a DST-NRF South African Research Chair grant (number 98406) awarded to JF. AR was a recipient of the Harold Crossley and NRF-Innovation PhD scholarships.

## Conflict of Interest

The authors declare that the research was conducted in the absence of any commercial or financial relationships that could be construed as a potential conflict of interest.

## Footnotes

1 In previous studies, plants collected from Buffelskloof Private Nature Reserve, Mpumalanga Province, South Africa, were classified at Xerophyta viscosa by Coetzee (1974). This population has been classified as Xerophyta schlechteri by Behnke et al. (2013). All references supplied in this article which worked from the Mpumalanga population, and thus on X. schlechteri, are asterisked in the reference section.

## Abbreviations

NST, Non-Senescent Tissue; ST, Senescent Tissue

## Supplementary Material

Supplementary Figure 1 Metabolite changes observed during desiccation and rehydration in the leaf tissues of Xerophyta schlechteri. ST (triangles) and NST (closed circles) leaf tissues were sampled from 10 adult X.schlechteri plants over the course of one dehydration-rehydration cycle. The metabolite concentrations were determined using various colorimetric assays and normalized the mass of the lyophilized starting tissue. Individual measurements were plotted against the recorded %RWC for each sample. Linear models were constructed using log transformed data and fitted to the untransformed data to product exponential models of changes in metabolite concentration as %RWC changes for the two tissue types. Model summary statistics in **Supplementary Table 1**. Graphics for changing total chlorophyll (A), total carotenoid (B), D-glucose (C), D-fructose (D) and sucrose (E) are depicted.

Supplementary figure 2 changes in amino acid accumulation during desiccation. Log2 transformed and pareto scaled normalized peak area of early-accumulating (60% RWC in NST) and late-accumulating (AD in NST) amino acids, detected by GC-MS. Error bars indicate standard deviation.

Supplementary figure 3 Changes in amino acid accumulation during desiccation (continued). Log2 transformed and pareto scaled normalized peak area of amino acids diminished in NST relative to FT NST and lysine (peaked at 35% RWC), detected by GC-MS. Error bars indicate standard deviation.

Supplementary figure 4 Changes in sugar accumulation during dehydration and rehydration. Log2 transformed and pareto scaled normalized peak area of sugars and sugar alcohols detected by GC-MS. Error bars indicate standard deviation.

Supplementary figure 5 Changes in organic acid and anti-oxidant accumulation during dehydration and rehydration Log2 transformed and pareto scaled normalized peak area of organic acids and anti-oxidants detected by GC-MS. Error bars indicate standard deviation.

Supplementary figure 6 Changes in uncategorized compound accumulation during dehydration and rehydration. Log2 transformed and pareto scaled normalized peak area of uncategorised compounds detected by GC-MS. Error bars indicate standard deviation.

Supplementary figure 7 Transmission electron micrographs of mesophyll during rehydration in ST. 48h RH ST (A-C) and 72h RH ST (D-F).

Supplementary Table 1 statistical test results for models in Supplementary figure 1. Significance codes: 0 ‘***’ 0,001 ‘**’ 0,01 ‘*’ 0,05 ‘.’ 0,1.

Supplementary Data Set 1 Ribitol-normalised metabolite peak area. Data is presented in ST relative to FT NST (tab 1, column 1), in NST relative to FT NST and in ST relative to NST at each sampling point (with NST RWC values displayed).
