# Supplementary figures and images for: Desiccation-Driven Senescence in the Resurrection Plant Xerophyta schlechteri (Baker) N.L. Menezes: Comparison of Anatomical, Ultrastructural, and Metabolic Responses Between Senescent and Non-Senescent Tissues
Source: Front Plant Sci. 2019 Oct 30;10:1396. doi: 10.3389/fpls.2019.01396 (PMC6831622; doi:10.3389/fpls.2019.01396)

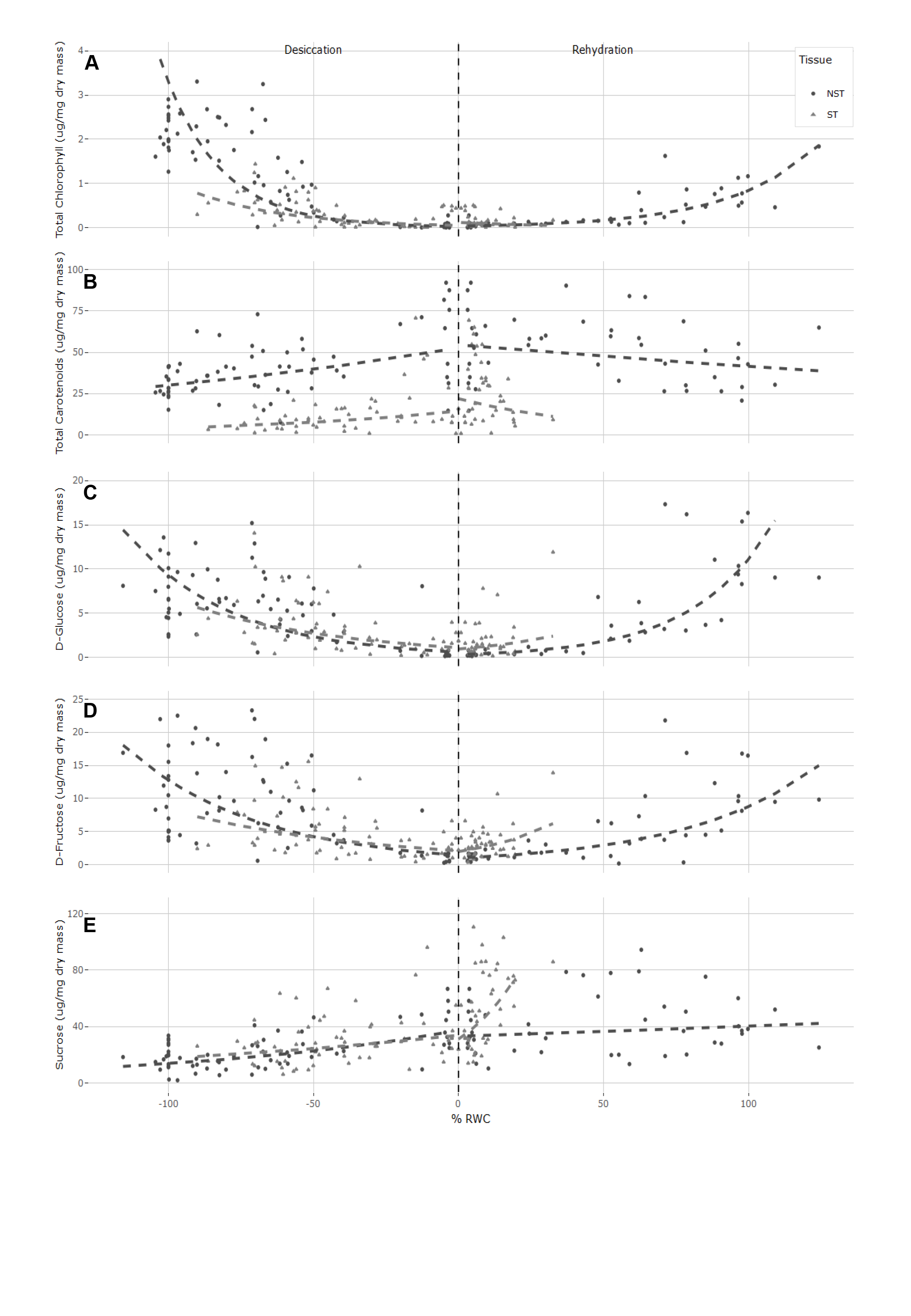

Supplement: Supplementary Figure 1 — Metabolite changes observed during desiccation and rehydration in the leaf tissues of Xerophyta schlechteri. ST (triangles) and NST (closed circles) leaf tissues were sampled from 10 adult X.schlechteri plants over the course of one dehydration-rehydration cycle. The metabolite concentrations were determined using various colorimetric assays and normalized the mass of the lyophilized starting tissue. Individual measurements were plotted against the recorded %RWC for each sample. Linear models were constructed using log transformed data and fitted to the untransformed data to product exponential models of changes in metabolite concentration as %RWC changes for the two tissue types. Model summary statistics in Supplementary Table 1 . Graphics for changing total chlorophyll (A), total carotenoid (B), D-glucose (C), D-fructose (D) and sucrose (E) are depicted. [file Image_1.png]

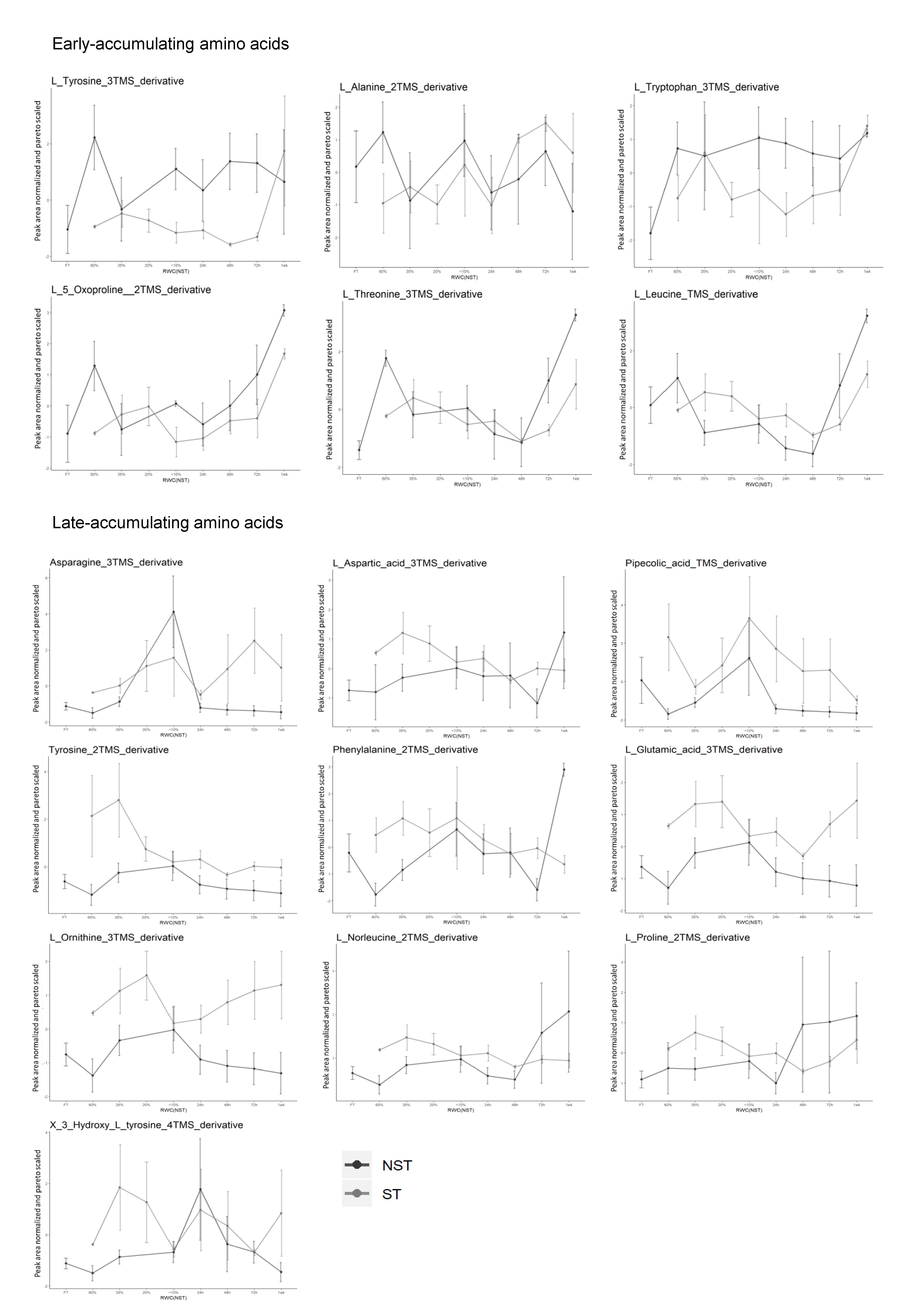

Supplement: Supplementary figure 2 — changes in amino acid accumulation during desiccation. Log2 transformed and pareto scaled normalized peak area of early-accumulating (60% RWC in NST) and late-accumulating (AD in NST) amino acids, detected by GC-MS. Error bars indicate standard deviation. [file Image_2.jpeg]

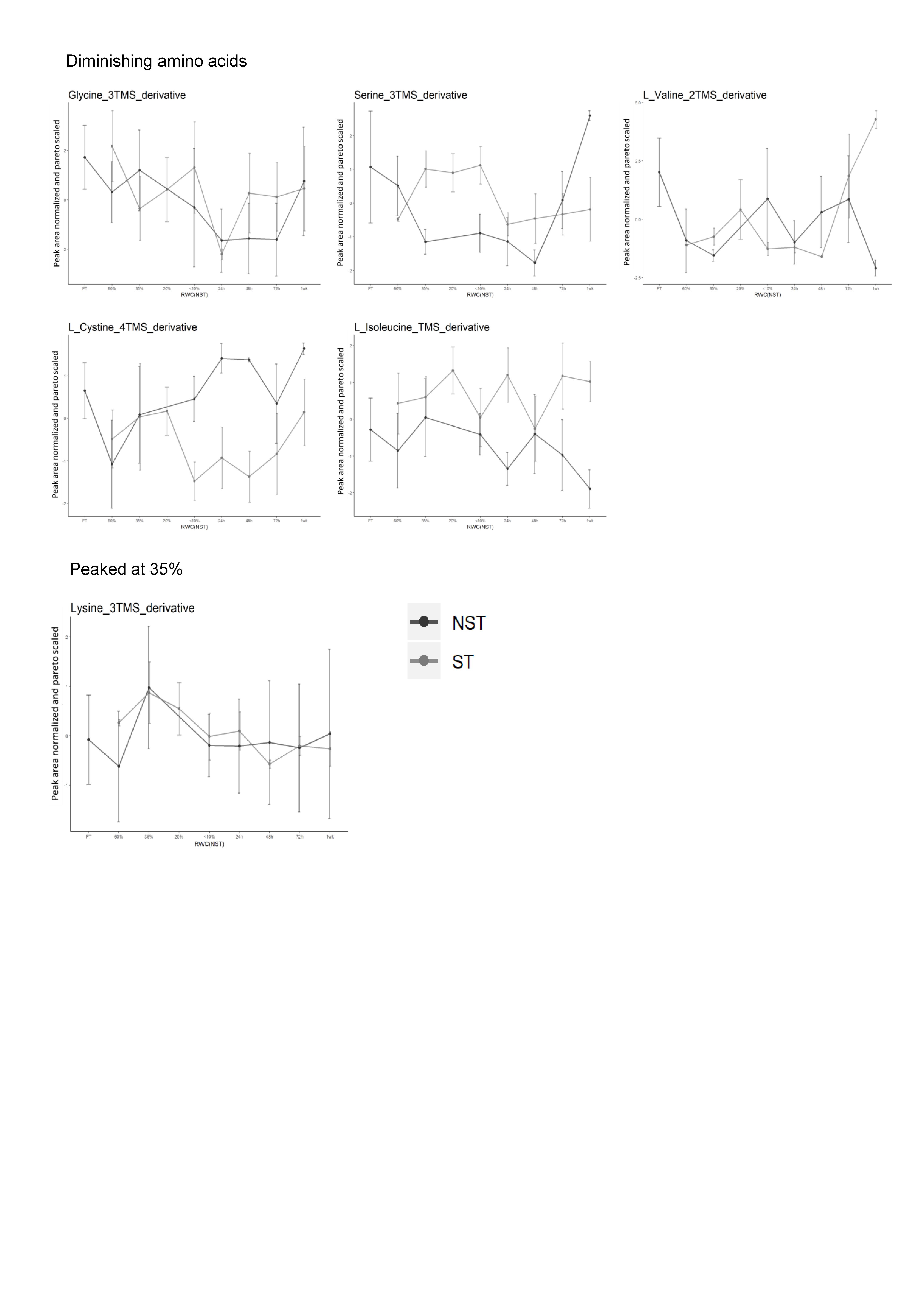

Supplement: Supplementary figure 3 — Changes in amino acid accumulation during desiccation (continued). Log2 transformed and pareto scaled normalized peak area of amino acids diminished in NST relative to FT NST and lysine (peaked at 35% RWC), detected by GC-MS. Error bars indicate standard deviation. [file Image_3.jpeg]

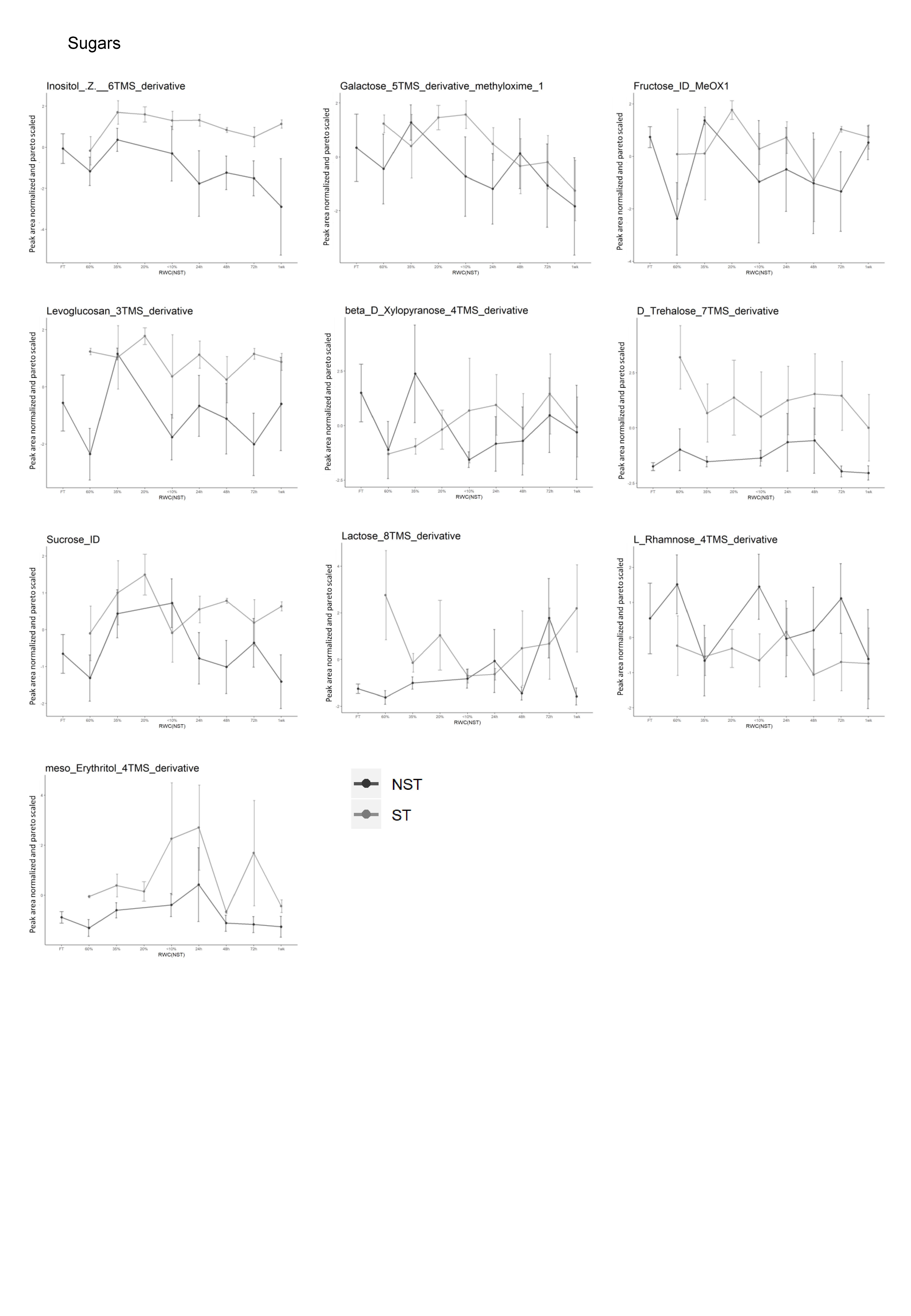

Supplement: Supplementary figure 4 — Changes in sugar accumulation during dehydration and rehydration. Log2 transformed and pareto scaled normalized peak area of sugars and sugar alcohols detected by GC-MS. Error bars indicate standard deviation. [file Image_4.jpeg]

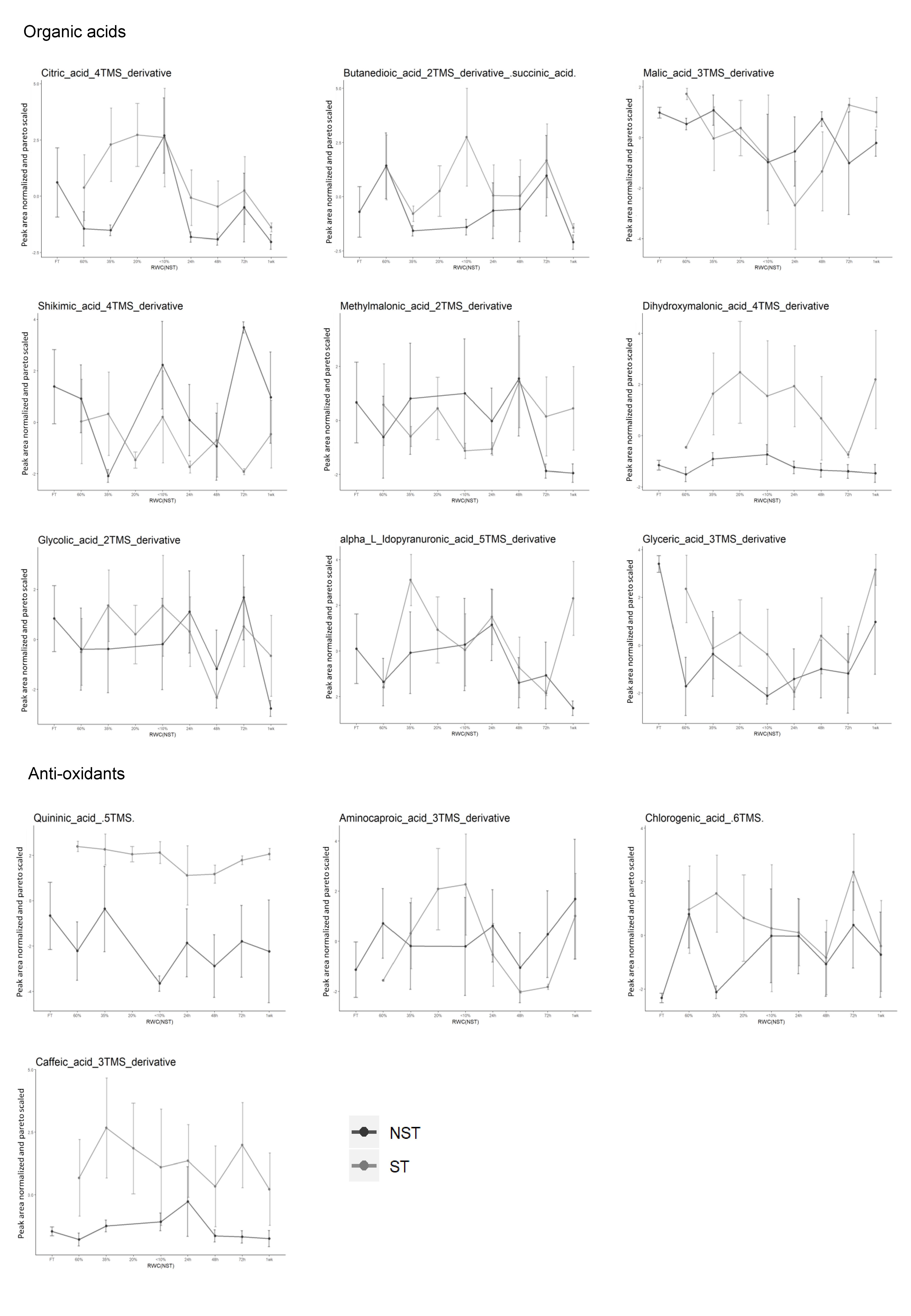

Supplement: Supplementary figure 5 — Changes in organic acid and anti-oxidant accumulation during dehydration and rehydration Log2 transformed and pareto scaled normalized peak area of organic acids and anti-oxidants detected by GC-MS. Error bars indicate standard deviation. [file Image_5.jpeg]

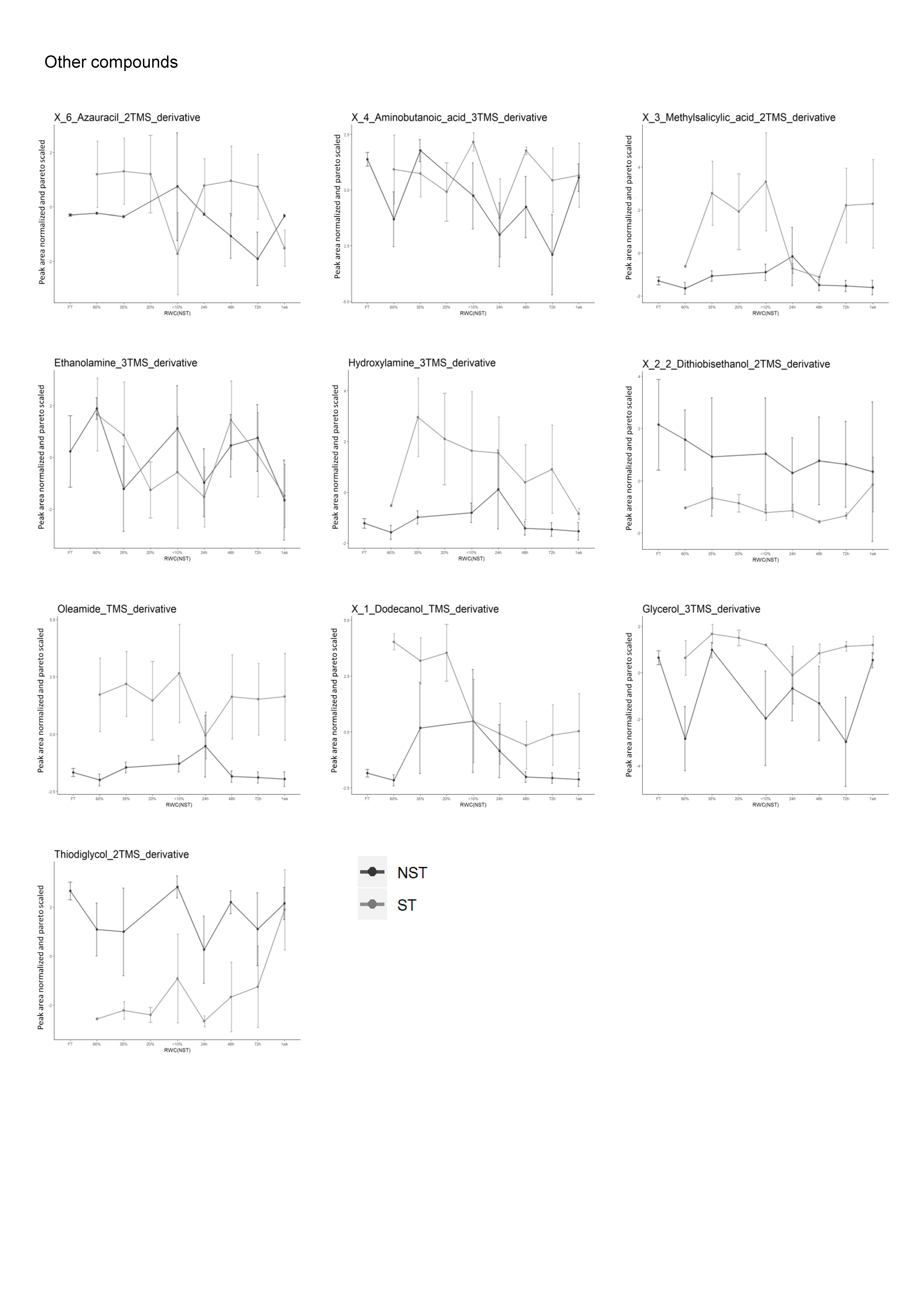

Supplement: Supplementary figure 6 — Changes in uncategorized compound accumulation during dehydration and rehydration. Log2 transformed and pareto scaled normalized peak area of uncategorised compounds detected by GC-MS. Error bars indicate standard deviation. [file Image_6.jpeg]

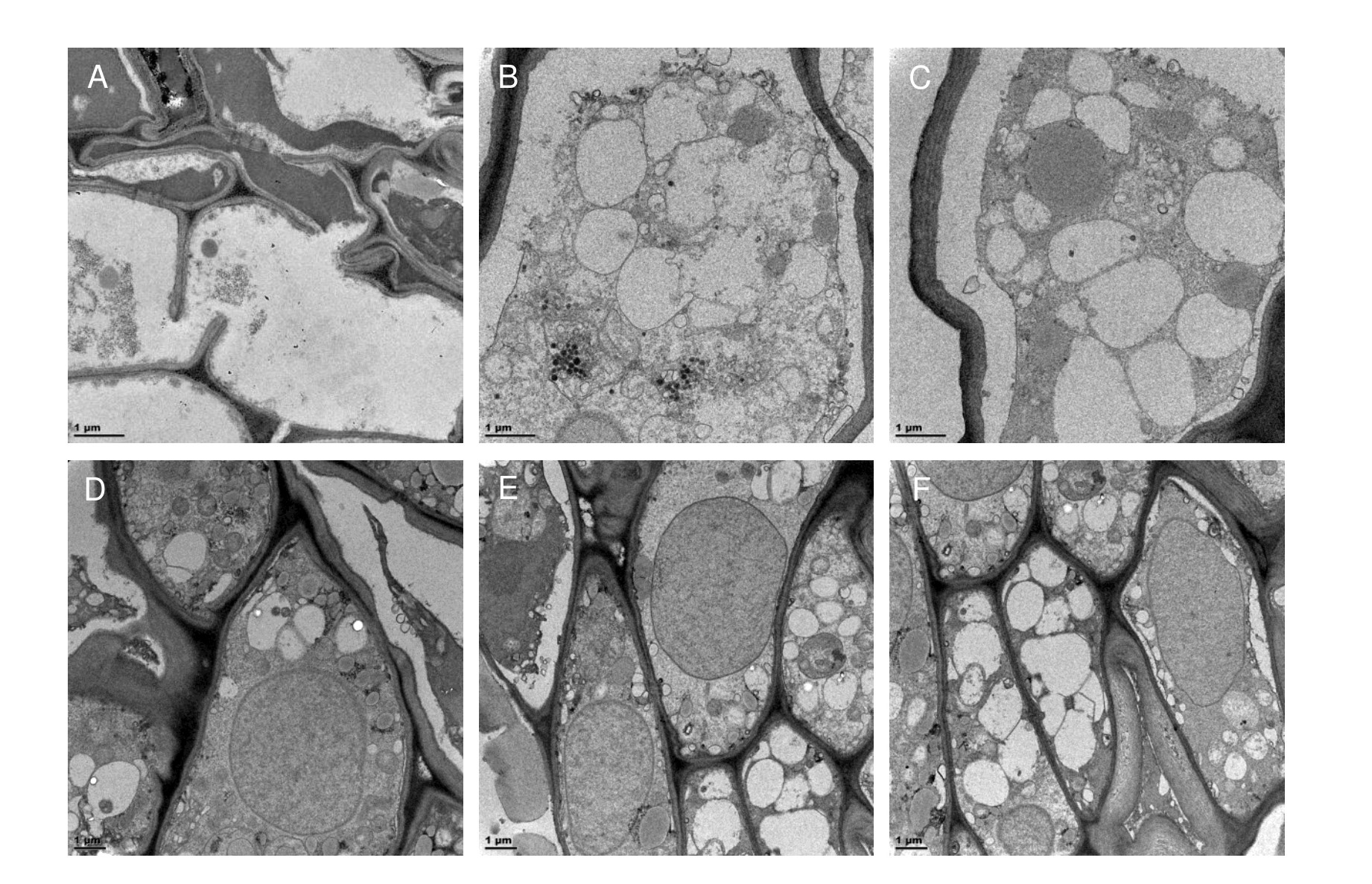

Supplement: Supplementary figure 7 — Transmission electron micrographs of mesophyll during rehydration in ST. 48h RH ST (A-C) and 72h RH ST (D-F). [file Image_7.jpeg]
