# Supplementary material for: Desiccation-Driven Senescence in the Resurrection Plant Xerophyta schlechteri (Baker) N.L. Menezes: Comparison of Anatomical, Ultrastructural, and Metabolic Responses Between Senescent and Non-Senescent Tissues
Source: Front Plant Sci. 2019 Oct 30;10:1396. doi: 10.3389/fpls.2019.01396 (PMC6831622; doi:10.3389/fpls.2019.01396)
Supplement: Supplementary Table 1 — statistical test results for models in Supplementary figure 1. Significance codes: 0 ‘***’ 0,001 ‘**’ 0,01 ‘*’ 0,05 ‘.’ 0,1. [file Table_1.pdf]

SUPPLEMENTARY TABLE1 | statistical test results for models in Supplementary figure 1.
Significance codes: 0 ‘***’ 0,001 ‘**’ 0,01 ‘*’ 0,05 ‘.’ 0,1.

A

Metabolite	Tissue	Residual St. Error	Df	Adjusted R^2	F	p	
Total Chlorophyll	NST	1.212	61	0.66	119.5	<0.001	***
	ST	1.345	56	0.21	16.53	<0.005	***
Total Carotenoids	NST	0.4319	61	0.15	11.52	<0.005	***
	ST	0.9025	51	0.08	5.731	0.02	*
D-Glucose	NST	0.8356	62	0.55	77.79	<0.001	***
	ST	0.8395	56	0.2	15.34	<0.001	***
D-Fructose	NST	0.8036	63	0.46	55.72	<0.001	***
	ST	0.7462	59	0.16	12.46	<0.001	***
Sucrose	NST	0.5766	62	0.25	21.58	<0.001	***
	ST	0.5566	59	0.06	4.836	0.032	*

B

Metabolite	Tissue	Residual St. Error	Df	Adjusted R^2	F	p	
Total Chlorophyll	NST	1.104	41	0.55	51.34	<0.001	***
	ST	1.137	33	<0.01	0.9089	0.347	
Total Carotenoids	NST	0.425	41	0.03	2.412	0.128	
	ST	0.9864	39	<0.01	0.7656	0.387	
D-Glucose	NST	0.6598	38	0.81	172.2	<0.001	***
	ST	0.9879	37	0.01	1.407	0.243	
D-Fructose	NST	0.8647	41	0.47	37.7	<0.001	***
	ST	0.647	40	0.09	5.167	0.028	*
Sucrose	NST	0.5315	41	<0.01	0.7738	0.384	
	ST	0.4849	41	0.25	14.99	<0.001	***
